# Supplementary material for: Connecting metal-organic framework synthesis to applications using multimodal machine learning
Source: Nat Commun. 2025 Jul 1;16:5642. doi: 10.1038/s41467-025-60796-0 (PMC12216699; doi:10.1038/s41467-025-60796-0)
Supplement: Supplementary file 1 — Supplementary Information [file 41467_2025_60796_MOESM1_ESM.pdf]

Connecting metal-organic framework synthesis to applications with multimodal machine learning

Sartaaj Takrim Khan and Seyed Mohamad Moosavi*

Chemical Engineering & Applied Chemistry, University of Toronto, Toronto, Ontario M5S 3E5, Canada

E-mail: mohamad.moosavi@utoronto.ca

Contents

1 MOF Databases **4**

2 Model architecture and hyperparameters **5**

3 Relevant property distributions **8**

3.1 CoRE-2019, ARABG-DB, BW20K-DB 8

3.2 QMOF 9

3.3 hMOF 10

4 CoRE-2019 filtration process **12**

5 Model performance **14**

5.1 Geometric properties 14

5.2 Chemistry-reliant and quantum chemical properties 14

5.3 Statistics of model performance 15
5.4 Statistics of benchmarked models 18

6 Recommendation system details 25
6.1 Threshold details 25
6.1.1 Justifications for thresholds 25
6.2 Masked loss function used 26

7 Statistics of recommendations system 28
7.1 Recommendations on computation-ready MOFs 28
7.2 Recommendations on Cambridge Structural Database (CSD) MOFs 34

8 "Time travel" model for MOF application discovery 37

9 Similarity Analysis of Time-Travel Sets 40

10 Evaluation of CoRE-MOF 2019's diversity 41

11 Model robustness on experimental PXRD patterns 46

12 Evaluation of model on covalent-organic frameworks 48

13 t-SNE map for MD/DFT labels 49

14 Data efficiency 51

15 Simulating powder x-ray diffraction patterns 54

16 Transformation of simulated powder x-ray diffraction patterns 55

17 Generation of MOF precursors 56

18 Powder x-ray diffraction pattern quality analysis 56

References **59**

1 MOF Databases

Table S1: Summary of metal-organic framework (MOF) databases used in this study. The number of used entries are the number of MOFs used on the machine learning (ML) side. This also showcases the different types of databases the model was assessed on (hypothetical vs. experimental). When referring to CoRE-2019-FILTERED, this is the original CoRE-2019[1] database but with duplicates and chemically infeasible MOFs removed, from utilizing MOFChecker.[2] Source data are provided as a Source Data file.

Database	Type of Database	Number of Used Entries
CoRE-2019[1,3]	Experimental	9,505
CoRE-2019-FILTERED	Experimental	3,904
BW20K-DB[3–5]	Hypothetical	12,673
ARABG-DB[3]	Hypothetical	350
QMOF[6]	Experimental	7,463
hMOF[3,7]	Hypothetical	5,320

Table S2: Training, validation, and test sizes along with the number of epochs for each database. It should be noted that during the preprocessing of powder x-ray diffraction (PXRD) patterns, the 2θ ranges from 0 to 40 degrees for all databases. Source data are provided as a Source Data file

Database	Train size	Validation size	Test size	Number of epochs
CoRE-MOF	3,319	390	585	100
BW20K	9,505	950	1,425	25
ARABG	175	87	87	100
hMOF	4,522	532	266	60
QMOF	6,343	746	373	60

2 Model architecture and hyperparameters

Table S3: The convolutional neural network (CNN) architecture and the respective hyperparameters in the architecture.[8] Source data are provided as a Source Data file.

Layer	Parameters
MaxPooling1D_1	kernel_size = 3
Conv1D_1	in_channels = 1, out_channels = 5, kernel_size = 3
ReLU_1	-
Conv1D_2	in_channels = 5, out_channels = 5, kernel_size = 3
ReLU_2	-
MaxPooling1D_2	kernel_size = 2
Conv1D_3	in_channels = 5, out_channels = 10, kernel_size = 3
ReLU_3	-
Conv1D_4	in_channels = 10, out_channels = 10, kernel_size = 3
ReLU_4	-
MaxPooling1D_3	kernel_size = 2
Conv1D_5	in_channels = 10, out_channels = 15, kernel_size = 5
ReLU_5	-
Conv1D_6	in_channels = 15, out_channels = 15, kernel_size = 5
ReLU_6	-
MaxPooling1D_4	kernel_size = 3
Conv1D_7	in_channels = 15, out_channels = 20, kernel_size = 5
ReLU_7	-
Conv1D_8	in_channels = 20, out_channels = 20, kernel_size = 5
ReLU_8	-
MaxPooling1D_5	kernel_size = 2
Conv1D_9	in_channels = 20, out_channels = 30, kernel_size = 5
ReLU_9	-
Conv1D_10	in_channels = 30, out_channels = 30, kernel_size = 5
ReLU_10	-
MaxPooling1D_6	kernel_size = 5
Flatten	-
Linear_1	in_features = 660, out_features = 80
ReLU_11	-
Linear_2	in_features = 80, out_features = 50
ReLU_12	-
Linear_3	in_features = 50, out_features = 10
ReLU_13	-
Linear_4	in_features = 10, out_features = 1
ReLU_14	-

The outputs from the convolutional neural network (CNN) (output shape: (N, 660) -

Table S4: Hyperparameters of the transformer utilized in our model framework - inspired from MOFormer's work,[9] in which a transformer accepted MOFids[10] to make property predictions. Source data are provided as a Source Data file.

**Hyperparameter**	**Value**
Embedding Dimension	512
Number of Heads	8
Dimension of Hidden Layers	512
Number of Layers	6
Dropout Rate	0.1
Number of Tokens	4021

model architecture given in Table S3) and the transformer (output shape: (N, 512, 512) - hyperparameters given in Table S4) are concatenated together over the 1st dimension. The resultant concatenated embedding is of shape (N, 1172), in which it is fed into a simple projector. The projector consists of a linear layer which projects the embedding from 1172 to 256, fed into a Softplus activation function and is then expanded to output a shape of (N, 512). This embedding from our concatenated model is used in self-supervised learning against the crystal graph convolutional neural network (CGCNN).[11]

The self-supervised learning framework (inspired from Crystal Twins framework[12]) was trained on batches of size 64, with both the CGCNN and the multimodal model having learning rates of 0.0005. The regularization parameter in Barlow-Twin,[13] λ, is initialized as 0.00051. The self-supervised learning was done over 100 epoch, with a train/validation split of 0.95/0.05. The learning curve of the SSL can be found in Figure S1.

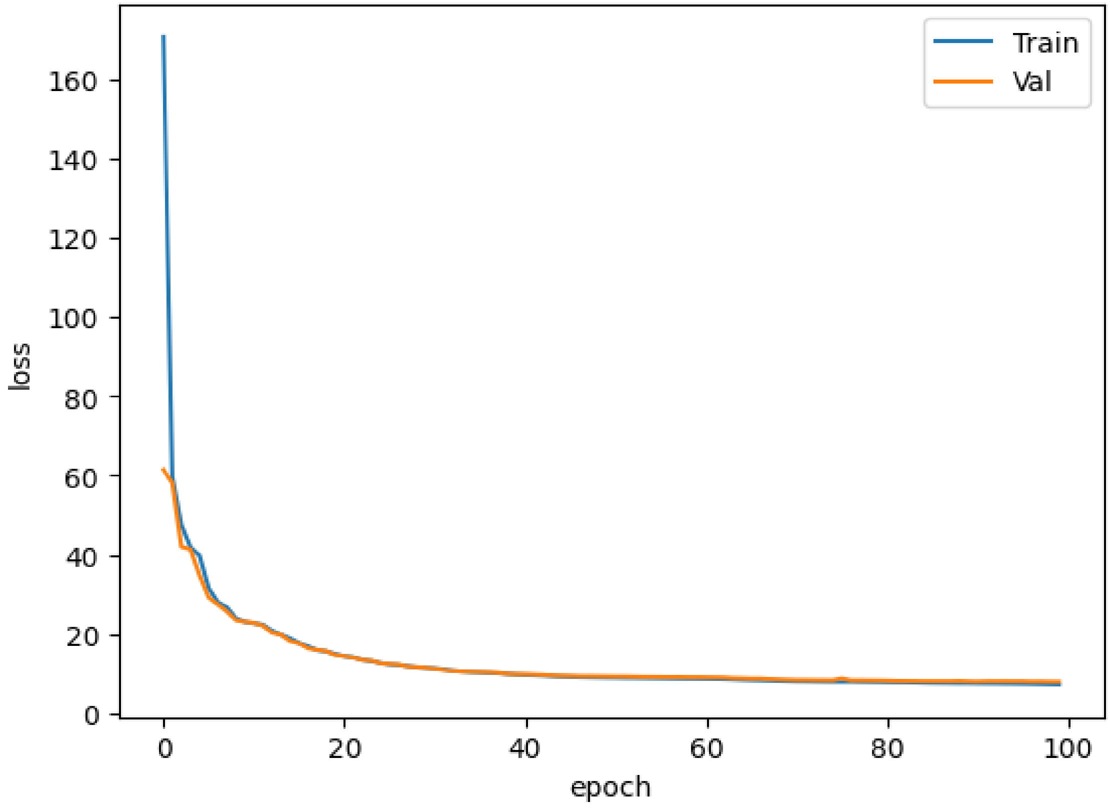

Figure S. 1| Learning curve showcasing Barlow-Twin loss against epochs elapsed between a crystal graph convolutional neural network (CGCNN) and our multimodal model, XRayPro. The train/val split taken here is 0.95/0.05. Source data are provided as a Source Data file.

3 Relevant property distributions

3.1 CoRE-2019, ARABG-DB, BW20K-DB

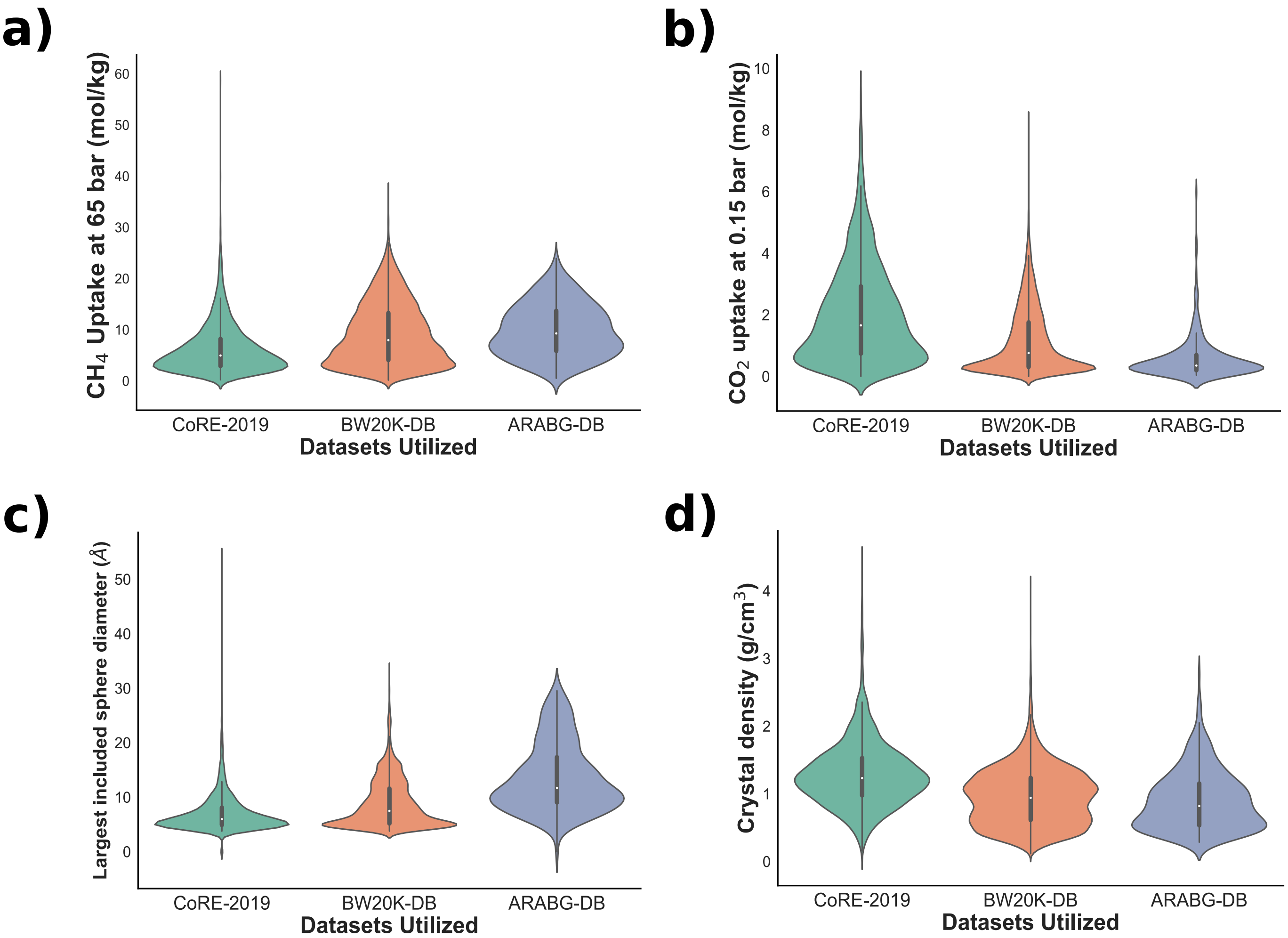

Figure S. 2| Relevant property distributions for various metal-organic framework (MOF) databases, including: CoRE-MOF 2019,[1] BW20K-DB,[4] ARABG-DB for (a) methane uptake at 65 bar, (b) carbon dioxide uptake at 0.15 bar, (c) largest included sphere diameter and (d) crystal density.[3] Source data are provided as a Source Data file.

3.2 QMOF

When initially loading the QMOF database, it has around 20,375 entries. However, out of these 20,375 entries, only 7,463 of them have a MOFid associated with it.[9] A comparison was done between the two distributions between the full QMOF-DB and the QMOF-DB used for training and model evaluation.

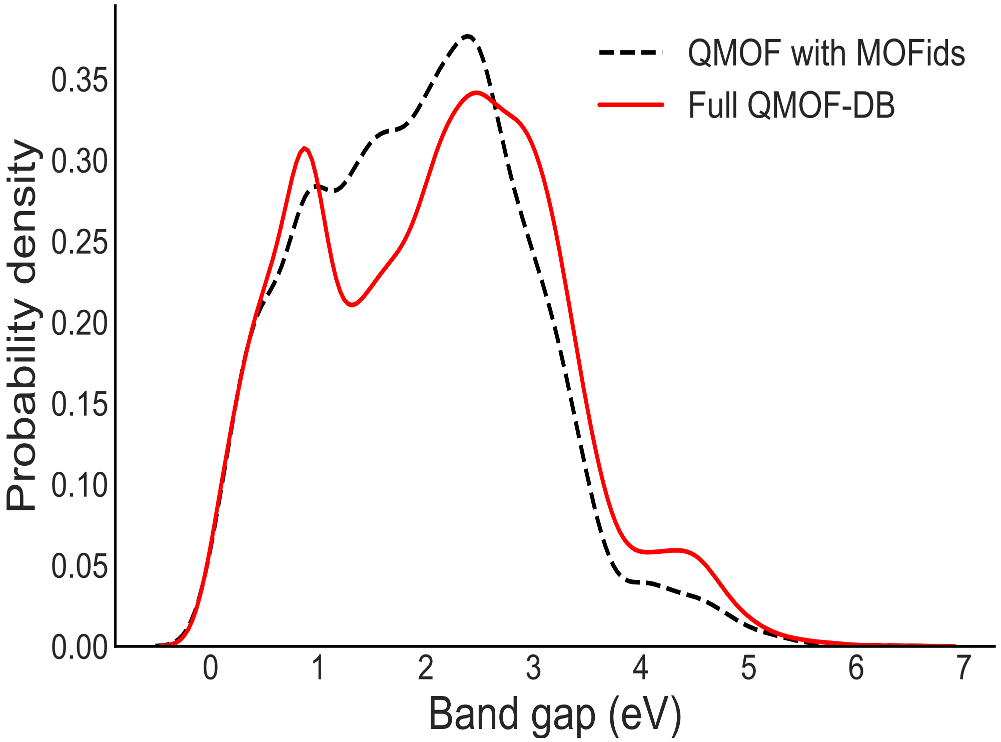

Figure S. 3| Probability distribution of metal-organic framework (MOF) band gap from the QMOF database.[6,14] As we only used the QMOF entries with a MOFid,[9,10] a comparison was done between the full QMOF database (solid red line) and the QMOF entries used (dashed line). Source data are provided as a Source Data file.

3.3 hMOF

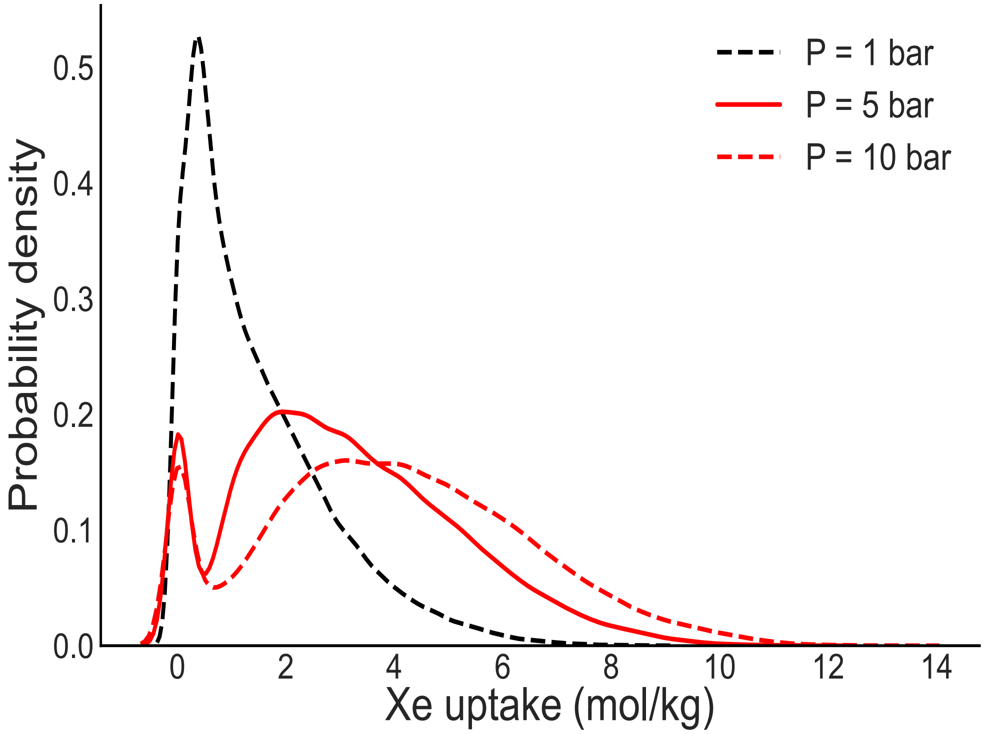

Figure S. 4| Probability distribution comparison between Xe uptake at varying pressures (1 bar, 5 bar, 10 bar) for metal-organic frameworks (MOFs) from the hMOF database.[7] Source data are provided as a Source Data file.

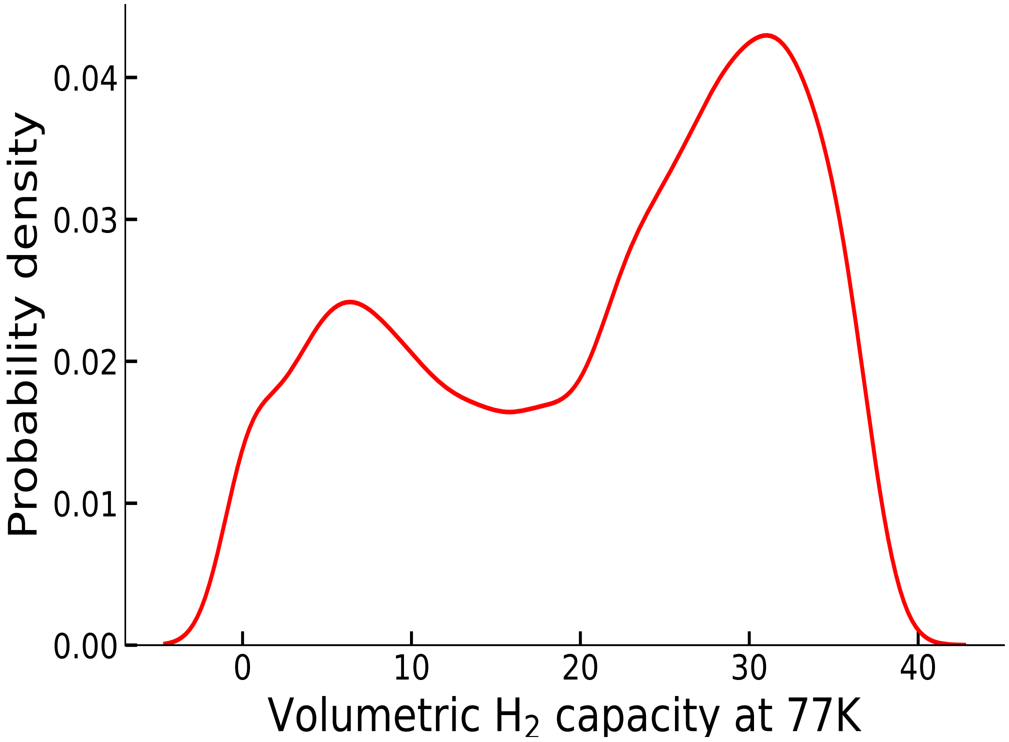

Figure S. 5| Probability distribution for H_2 capacity at 77K for a pressure swing between 100 to 5 bars for metal-organic frameworks (MOFs) from the hMOF database.[7] Source data are provided as a Source Data file.

4 CoRE-2019 filtration process

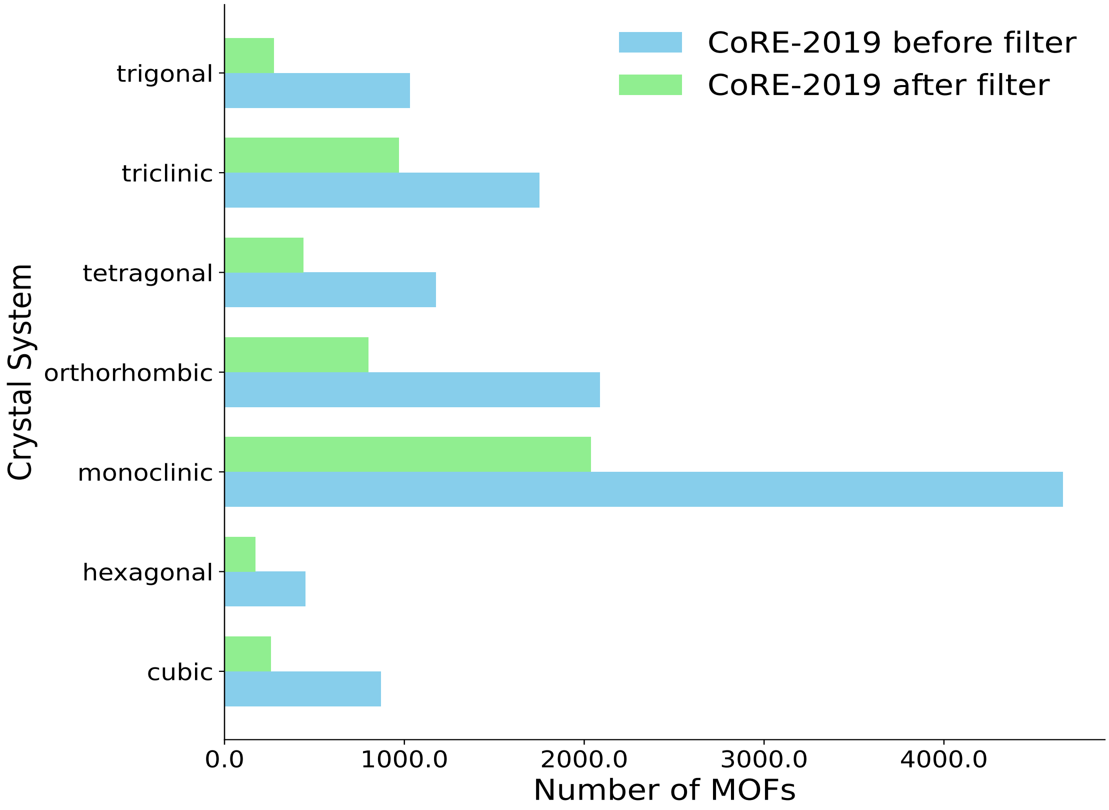

Figure S.6| Breakdown of CoRE-2019[1] crystal systems before and after the filtration process described above. This showcases that, despite the database filtration, the distribution of crystal systems before and after the filter are relatively similar. Source data are provided as a Source Data file.

The CoRE-MOF database required cleaning, as previous work has shown the presence of duplicates and chemically infeasible structure.[1,15] Due to this, we decided to run "sanity checks" on the CoRE-MOF 2019 database to remove duplicates and MOFs that are not possible. The criteria for an "invalid MOF" is if they do not have carbon atoms, hydrogen atoms, metal nodes, contains atomic overlaps, contains carbon atoms with more than four neighbours or does not contain open metal sites. Initially, we start with 12,007 CoRE-MOF entries. Through using MOFChecker[2] to run the sanity checks, we screened through the CoRE-MOF 2019 database and ended with 6,027 MOFs. Afterwards, the structures from

the 6,027 MOFs were hashed, as each unique molecular graph returns a unique hash. From performing a set function, the unique hashes are returned, which returns a database size of 4,944. Finally, the MOFs without a valid MOFid[10] were removed and returned a final CoRE-MOF 2019 database of 3,904.

5 Model performance

5.1 Geometric properties

A series of geometric properties were predicted: methane uptake at 65 bar, crystal density, pore diameter (CoRE,[1,3] BW20K,[3,4] ARABG[3]), hydrogen storage capacity and xenon uptake (hMOF) at varying pressures. A comparison between the scratch and pretrained model were done to evaluate the model performance, although it should be noted that minor improvement was made due to the model performance for geometric properties already being good and the PXRD seemingly able to capture the global environments of the MOF. Furthermore, this was on high data regimes.

5.2 Chemistry-reliant and quantum chemical properties

Chemistry-reliant and quantum chemical properties were predicted as an assessment on how flexible the model is on labels that are not geometric. Furthermore, it gives us an idea of how much information the chemical precursors can provide a deep learning model on the metal and organic chemistry of a MOF. In this case, the chemistry-reliant property is carbon dioxide uptake at 0.15 bar (labels taken from CoRE-MOF, BW20K-DB, ARABG-DB), and the quantum chemical property is band gap (labels taken from QMOF).[6]

5.3 Statistics of model performance

Table S5: Comparison of our scratch and pretrained models on the metal-organic framework (MOF) database CoRE-MOF[1,3] across n = 3 random seeds. Geometric (CH_4 uptake at HP - high pressure, pore diameter, density, logK_H, CH_4 DC - deliverable capacity) and chemistry-reliant properties (CO_2 uptake at LP - low pressure) were used for this evaluation. The units for CH_4 uptake at HP, CO_2 uptake at LP, pore diameter, density and CH_4 DC are mol/kg, mol/kg, Å, g/cm^3 and v STP/v respectively. logK_H is unitless, in this case. The dataset and split sizes can be found in Table S2. The scores in the table are the mean and standard deviation values across the randomized seeds. Source data are provided as a Source Data file.

**Property**	**MAE**	**MSE**	**RMSE**	**SRCC**
		**Scratch Model**		
CH_4 uptake @ HP	1.39 ± 0.04	4.25 ± 0.53	2.06 ± 0.12	0.89 ± 0.01
CO_2 uptake @ LP	0.87 ± 0.03	1.46 ± 0.12	1.21 ± 0.05	0.67 ± 0.02
Pore diameter	1.08 ± 0.05	3.49 ± 0.16	1.86 ± 0.04	0.82 ± 0.01
Density	0.13 ± 0.004	0.04 ± 0.002	0.19 ± 0.005	0.92 ± 0.01
LogKH_CO_2	0.61 ± 0.03	0.66 ± 0.02	0.81 ± 0.01	0.70 ± 0.01
CH_4 DC	25.2 ± 1.23	1112 ± 96.0	33.3 ± 1.44	0.77 ± 0.01
		**Pretrained Model**		
CH_4 uptake @ HP	1.35 ± 0.02	3.78 ± 0.16	1.94 ± 0.04	0.90 ± 0.01
CO_2 uptake @ LP	0.84 ± 0.02	1.41 ± 0.06	1.18 ± 0.03	0.69 ± 0.02
Pore diameter	1.15 ± 0.11	4.02 ± 0.90	1.99 ± 0.22	0.82 ± 0.01
Density	0.13 ± 0.004	0.036 ± 0.002	0.19 ± 0.006	0.91 ± 0.01
LogKH_CO_2	0.57 ± 0.02	0.62 ± 0.03	0.78 ± 0.02	0.72 ± 0.02
CH_4 DC	24.4 ± 0.59	1062 ± 68.8	32.6 ± 0.01	0.78 ± 0.01

Table S6: Comparison of our scratch and pretrained models on the metal-organic framework (MOF) database BW20K[3,4] across n = 3 random seeds. Geometric (CH_4 uptake at HP - high pressure, pore diameter, density, logK_H) and chemistry-reliant properties (CO_2 uptake at LP - low pressure) were used for this evaluation. The units for CH_4 uptake at HP, CO_2 uptake at LP, pore diameter and density are mol/kg, mol/kg, Åand g/cm^3 respectively. logK_H is unitless, in this case. The dataset and split sizes can be found in Table S2. The scores in the table are the mean and standard deviation values across the randomized seeds. Source data are provided as a Source Data file.

**Property**	**MAE**	**MSE**	**RMSE**	**SRCC**
**Scratch Model**				
CH_4 uptake @ HP	0.97 ± 0.04	1.67 ± 0.15	1.29 ± 0.06	0.97 ± 0.00
CO_2 uptake @ LP	0.39 ± 0.05	0.33 ± 0.09	0.57 ± 0.07	0.84 ± 0.04
Pore diameter	0.86 ± 0.05	1.53 ± 0.19	1.23 ± 0.07	0.95 ± 0.00
Density	0.07 ± 0.01	0.01 ± 0.00	0.09 ± 0.02	0.97 ± 0.01
LogKH_CO_2	0.25 ± 0.01	0.13 ± 0.01	0.35 ± 0.01	0.88 ± 0.00
**Pretrained Model**				
CH_4 uptake @ HP	0.76 ± 0.01	1.05 ± 0.03	1.02 ± 0.01	0.98 ± 0.00
CO_2 uptake @ LP	0.31 ± 0.00	0.21 ± 0.00	0.46 ± 0.00	0.90 ± 0.00
Pore diameter	0.72 ± 0.01	1.08 ± 0.04	1.04 ± 0.02	0.97 ± 0.00
Density	0.05 ± 0.00	0.01 ± 0.00	0.07 ± 0.00	0.99 ± 0.00
LogKH_CO_2	0.23 ± 0.00	0.10 ± 0.01	0.33 ± 0.01	0.90 ± 0.01

Table S7: Comparison of our scratch and pretrained models on the metal-organic framework (MOF) database ARABG[3] across n = 3 random seeds. Geometric (CH_4 uptake at HP - high pressure, pore diameter, density, logK_H) and chemistry-reliant properties (CO_2 uptake at LP - low pressure) were used for this evaluation. The units for CH_4 uptake at HP, CO_2 uptake at LP, pore diameter and density are mol/kg, mol/kg, Åand g/cm^3 respectively. logK_H is unitless, in this case. The dataset and split sizes can be found in Table S2. The scores in the table are the mean and standard deviation values across the randomized seeds. Source data are provided as a Source Data file.

**Property**	**MAE**	**MSE**	**RMSE**	**SRCC**
		**Scratch Model**		
CH_4 uptake @ HP	1.24 ± 0.15	3.64 ± 1.67	1.86 ± 0.42	0.92 ± 0.03
CO_2 uptake @ LP	0.32 ± 0.06	0.28 ± 0.09	0.52 ± 0.08	0.64 ± 0.05
Pore diameter	1.91 ± 0.20	6.54 ± 0.80	2.55 ± 0.16	0.90 ± 0.03
Density	0.14 ± 0.04	0.04 ± 0.02	0.18 ± 0.05	0.87 ± 0.09
LogKH_CO_2	0.26 ± 0.02	0.11 ± 0.02	0.33 ± 0.03	0.76 ± 0.07
		**Pretrained Model**		
CH_4 uptake @ HP	1.11 ± 0.13	2.31 ± 0.56	1.51 ± 0.18	0.95 ± 0.01
CO_2 uptake @ LP	0.22 ± 0.03	0.14 ± 0.06	0.37 ± 0.07	0.83 ± 0.01
Pore diameter	1.20 ± 0.12	3.24 ± 1.62	1.75 ± 0.43	0.95 ± 0.01
Density	0.11 ± 0.01	0.02 ± 0.001	0.13 ± 0.004	0.94 ± 0.001
LogKH_CO_2	0.19 ± 0.01	0.07 ± 0.01	0.26 ± 0.03	0.84 ± 0.03

Table S8: Comparison of our scratch and pretrained models on the metal-organic framework (MOF) databases of QMOF (band gap)[6,14] and hMOF[7] (hydrogen capacity and Xe uptake at HP - high pressure). For QMOF, it was run over n = 3 random seeds, whereas hMOF was run over 2 random seeds (due to computational restrictions). The scores given are the mean and standard deviation values across the randomized seeds. The units for band gap, H_2 capacity and Xe uptake at HP are eV, g/L and mol/kg respectively. Source data are provided as a Source Data file.

**Property**	**MAE**	**MSE**	**RMSE**	**SRCC**
		**Scratch Model**		
Band gap	0.39 ± 0.02	0.28 ± 0.03	0.53 ± 0.03	0.85 ± 0.01
H_2 Capacity	1.88 ± 0.07	6.13 ± 0.58	2.47 ± 0.12	0.96 ± 0.00
Xe uptake @ HP	0.58 ± 0.03	0.61 ± 0.03	0.78 ± 0.02	0.95 ± 0.00
		**Pretrained Model**		
Band gap	0.38 ± 0.01	0.26 ± 0.03	0.51 ± 0.03	0.87 ± 0.004
H_2 Capacity	1.54 ± 0.06	4.31 ± 0.06	2.08 ± 0.01	0.98 ± 0.00
Xe uptake @ HP	0.56 ± 0.00	0.58 ± 0.00	0.76 ± 0.00	0.95 ± 0.00

5.4 Statistics of benchmarked models

Table S9: Performance metrics for a crystal graph convolutional neural network (CGCNN) [11] across various geometric (CH_4 uptake at HP - high pressure, CO_2 uptake at LP - low pressure, H_2 capacity, Xe uptake at HP, pore diameter, accessible surface area - ASA, density, CH_4 DC - deliverable capacity), chemistry-reliant (CO_2 uptake at LP - low pressure) and quantum-chemical (band gap) properties for various metal-organic framework (MOF) databases. The units for gas uptake, band gap, H_2 capacity, pore diameter, ASA density and deliverable capacity are mol/kg, eV, g/L, Angstrom, m^2/cm^3, g/cm^3 and vSTP/v respectively. This was run over n = 3 random seeds, with the scores reported being the mean and standard deviation across these trials. The database utilized for CH_4 uptake at HP, CO_2 uptake at LP, pore diameter, ASA, density and CH_4 DC is CoRE-2019.[1,3] The database utilized for band gap is QMOF.[6,14] The database utilized for H_2 capacity and Xe uptake at HP is hMOF.[7] Source data are provided as a Source Data file.

**Property**	**MAE**	**MSE**	**RMSE**	**SRCC**
CH_4 uptake at HP	1.52 ± 0.04	4.17 ± 0.26	2.04 ± 0.06	0.82 ± 0.01
CO_2 uptake at LP	0.73 ± 0.006	1.02 ± 0.05	1.01 ± 0.03	0.77 ± 0.002
Band gap	0.34 ± 0.009	0.22 ± 0.006	0.46 ± 0.006	0.88 ± 0.001
H_2 capacity	2.51 ± 0.08	11.95 ± 0.19	3.45 ± 0.03	0.92 ± 0.006
Xe uptake at HP	1.02 ± 0.02	1.72 ± 0.008	1.31 ± 0.003	0.83 ± 0.001
Pore diameter	1.68 ± 0.01	6.74 ± 0.07	2.59 ± 0.01	0.61 ± 0.01
ASA	410.4 ± 11.8	283966 ± 9459	532 ± 8.8	0.74 ± 0.01
Density	0.12 ± 0.004	0.03 ± 0.002	0.16 ± 0.01	0.91 ± 0.01
CH_4 DC	25.5 ± 0.46	1103 ± 46.4	33.2 ± 0.69	0.77 ± 0.01

Table S10: Performance metrics for a transformer-based model (MOFormer)[9] that accepts a string-based representation of a metal-organic framework (MOF), in the form of the inorganic metal cluster, the organic linker, the topological code and the catenation (MOFid).[10] This was done across various geometric (CH_4 uptake at HP - high pressure, CO_2 uptake at LP - low pressure, H_2 capacity, Xe uptake at HP, pore diameter, accessible surface area - ASA, density, CH_4 DC - deliverable capacity), chemistry-reliant (CO_2 uptake at LP - low pressure) and quantum-chemical (band gap) properties for various MOF databases. The units for gas uptake, band gap, H_2 capacity, pore diameter, ASA, density and deliverable capacity are mol/kg, eV, g/L, Å, m^2/cm^3, g/cm^3 and vSTP/v respectively. This was run over n = 3 random seeds, with the scores reported being the mean and standard deviation across these trials. The database utilized for CH_4 uptake at HP, CO_2 uptake at LP, pore diameter, ASA, density and CH_4 DC is CoRE-2019.[1,3] The database utilized for band gap is QMOF.[6,14] The database utilized for H_2 capacity and Xe uptake at HP is hMOF.[7] Source data are provided as a Source Data file.

**Property**	**MAE**	**MSE**	**RMSE**	**SRCC**
CH_4 uptake at HP	2.47 ± 0.08	12.72 ± 1.28	3.56 ± 0.18	0.61 ± 0.02
CO_2 uptake at LP	0.95 ± 0.01	1.78 ± 0.01	1.33 ± 0.01	0.57 ± 0.03
Band gap	0.36 ± 0.003	0.25 ± 0.003	0.50 ± 0.003	0.87 ± 0.003
H_2 capacity	3.04 ± 0.07	21.55 ± 1.49	4.63 ± 0.16	0.90 ± 0.01
Xe uptake at HP	0.92 ± 0.002	1.70 ± 0.002	1.30 ± 0.00	0.84 ± 0.003
Pore diameter	1.75 ± 0.04	7.27 ± 0.58	2.69 ± 0.11	0.49 ± 0.02
ASA	574.1 ± 18.3	574021 ± 55376	756.7 ± 36.1	0.46 ± 0.02
Density	0.22 ± 0.01	0.09 ± 0.001	0.30 ± 0.002	0.73 ± 0.01
CH_4DC	36.53 ± 0.86	2291.5 ± 183.9	47.8 ± 1.94	0.44 ± 0.02

Table S11: Performance metrics for an XGBoost model accepting RACs and geometric descriptors for metal-organic frameworks (MOFs).[3] This was done across various geometric (CH_4 uptake at HP - high pressure, CO_2 uptake at LP - low pressure, H_2 capacity, Xe uptake at HP, pore diameter, accessible surface area - ASA, density, CH_4 DC - deliverable capacity), chemistry-reliant (CO_2 uptake at LP - low pressure) and quantum-chemical (band gap) properties for various MOF databases. The units for gas uptake, band gap, H_2 capacity, pore diameter, ASA, density and deliverable capacity are mol/kg, eV, g/L, Å, m^2/cm^3, g/cm^3 and vSTP/v respectively. This was run over n = 3 random seeds, with the scores reported being the mean and standard deviation across these trials. The database utilized for CH_4 uptake at HP, CO_2 uptake at LP, pore diameter, ASA, density and CH_4 DC is CoRE-2019.[1,3] The database utilized for band gap is QMOF.[6,14] The database utilized for H_2 capacity and Xe uptake at HP is hMOF.[7] Source data are provided as a Source Data file.

**Property**	**MAE**	**MSE**	**RMSE**	**SRCC**
CH_4 uptake at HP	0.45 ± 0.01	0.55 ± 0.12	0.73 ± 0.08	0.98 ± 0.00
CO_2 uptake at LP	0.54 ± 0.01	0.65 ± 0.03	0.81 ± 0.02	0.83 ± 0.001
Band gap	0.41 ± 0.03	0.38 ± 0.04	0.62 ± 0.03	0.84 ± 0.008
H_2 capacity	0.76 ± 0.01	1.15 ± 0.01	1.07 ± 0.003	0.99 ± 0.00
Xe uptake at HP	0.45 ± 0.004	0.42 ± 0.01	0.65 ± 0.008	0.96 ± 0.0008
Pore diameter	0.045 ± 0.01	0.24 ± 0.15	0.46 ± 0.17	0.99 ± 0.00
ASA	2.87 ± 0.15	59.09 ± 22.71	7.54 ± 1.49	0.97 ± 0.002
Density	0.0042 ± 0.00	0.00036 ± 0.00	0.02 ± 0.004	0.99 ± 0.00
CH_4DC	10.62 ± 0.27	245.1 ± 15.6	15.64 ± 0.51	0.94 ± 0.007

Table S12: Performance metrics for a transformer only accepting metal-organic framework (MOF) precursors, in the form of [metal type].[SMILES of the organic linker], as opposed to the MOFid[10] which contains information about the metal cluster, topological code and degree of catenation. This was done across various geometric (CH_4 uptake at HP - high pressure, CO_2 uptake at LP - low pressure, H_2 capacity, Xe uptake at HP, pore diameter, accessible surface area - ASA, density, CH_4 DC - deliverable capacity), chemistry-reliant (CO_2 uptake at LP - low pressure) and quantum-chemical (band gap) properties for various MOF databases. The units for gas uptake, band gap, H_2 capacity, pore diameter, ASA, density and deliverable capacity are mol/kg, eV, g/L, Å, m^2/cm^3, g/cm^3 and vSTP/v respectively. This was run over $n = 3$ random seeds, with the scores reported being the mean and standard deviation across these trials. The database utilized for CH_4 uptake at HP, CO_2 uptake at LP, pore diameter, ASA, density and CH_4 DC is CoRE-2019.[1,3] The database utilized for band gap is QMOF.[6,14] The database utilized for H_2 capacity and Xe uptake at HP is hMOF.[7] Source data are provided as a Source Data file.

**Property**	**MAE**	**MSE**	**RMSE**	**SRCC**
CH_4 uptake at HP	2.53 ± 0.11	13.37 ± 1.30	3.65 ± 0.17	0.61 ± 0.01
CO_2 uptake at LP	1.00 ± 0.03	1.84 ± 0.07	1.35 ± 0.03	0.53 ± 0.03
Band gap	0.41 ± 0.003	0.31 ± 0.002	0.56 ± 0.002	0.84 ± 0.002
H_2 capacity	7.46 ± 0.002	86.87 ± 1.72	9.32 ± 0.09	0.47 ± 0.002
Xe uptake at HP	1.44 ± 0.02	3.27 ± 0.02	1.81 ± 0.02	0.66 ± 0.01
Pore diameter	1.99 ± 0.03	10.41 ± 0.93	3.22 ± 0.14	0.39 ± 0.03
ASA	604.60 ± 3.72	545711 ± 10513	738.6 ± 7.10	0.43 ± 0.02
Density	0.23 ± 0.002	0.09 ± 0.002	0.31 ± 0.002	0.70 ± 0.002
CH_4DC	38.4 ± 1.29	2488.5 ± 2.23	49.88 ± 0.02	0.43 ± 0.01

Table S13: Performance metrics for a convolutional neural network (CNN)[8] only accepting powder x-ray diffraction (PXRD) patterns of metal-organic frameworks (MOFs). This was done across various geometric (CH_4 uptake at HP - high pressure, CO_2 uptake at LP - low pressure, H_2 capacity, Xe uptake at HP, pore diameter, accessible surface area - ASA, density, CH_4 DC - deliverable capacity), chemistry-reliant (CO_2 uptake at LP - low pressure) and quantum-chemical (band gap) properties for various MOF databases. The units for gas uptake, band gap, H_2 capacity, pore diameter, ASA, density and deliverable capacity are mol/kg, eV, g/L, Å, m^2/cm^3, g/cm^3 and vSTP/v respectively. This was run over n = 3 random seeds, with the scores reported being the mean and standard deviation across these trials. The database utilized for CH_4 uptake at HP, CO_2 uptake at LP, pore diameter, ASA, density and CH_4 DC is CoRE-2019.[1,3] The database utilized for band gap is QMOF.[6,14] The database utilized for H_2 capacity and Xe uptake at HP is hMOF.[7] Source data are provided as a Source Data file.

**Property**	**MAE**	**MSE**	**RMSE**	**SRCC**
CH_4 uptake at HP	1.72 ± 0.04	5.96 ± 0.49	2.44 ± 0.09	0.83 ± 0.003
CO_2 uptake at LP	1.07 ± 0.05	2.07 ± 0.05	1.43 ± 0.06	0.48 ± 0.01
Band gap	0.75 ± 0.03	0.89 ± 0.07	0.94 ± 0.04	0.36 ± 0.04
H_2 capacity	2.39 ± 0.08	10.30 ± 0.50	3.21 ± 0.07	0.95 ± 0.004
Xe uptake at HP	0.80 ± 0.01	1.21 ± 0.02	1.09 ± 0.01	0.90 ± 0.0003
Pore diameter	1.15 ± 0.03	3.61 ± 0.56	1.89 ± 0.15	0.79 ± 0.02
ASA	435.6 ± 27.5	327156 ± 35586	571.1 ± 31.5	0.71 ± 0.04
Density	0.16 ± 0.01	0.05 ± 0.003	0.23 ± 0.01	0.87 ± 0.01
CH_4DC	28.5 ± 0.50	1396.6 ± 25.5	37.4 ± 0.34	0.72 ± 0.001

Table S14: Performance metrics for our model, which accepts the powder x-ray diffraction (PXRD) pattern and the chemical precursors of a metal-organic framework (MOF).This was done across various geometric (CH_4 uptake at HP - high pressure, CO_2 uptake at LP - low pressure, H_2 capacity, Xe uptake at HP, pore diameter, accessible surface area - ASA, density, CH_4 DC - deliverable capacity), chemistry-reliant (CO_2 uptake at LP - low pressure) and quantum-chemical (band gap) properties for various MOF databases. The units for gas uptake, band gap, H_2 capacity, pore diameter, ASA, density and deliverable capacity are mol/kg, eV, g/L, Å, m^2/cm^3, g/cm^3 and vSTP/v respectively. This was run over n = 3 random seeds, with the scores reported being the mean and standard deviation across these trials. The database utilized for CH_4 uptake at HP, CO_2 uptake at LP, pore diameter, ASA, density and CH_4 DC is CoRE-2019.[1,3] The database utilized for band gap is QMOF.[6,14] The database utilized for H_2 capacity and Xe uptake at HP is hMOF.[7] Source data are provided as a Source Data file.

**Property**	**MAE**	**MSE**	**RMSE**	**SRCC**
CH_4 uptake at HP	1.35 ± 0.02	3.78 ± 0.16	1.94 ± 0.04	0.89 ± 0.01
CO_2 uptake at LP	0.84 ± 0.02	1.41 ± 0.06	1.18 ± 0.03	0.69 ± 0.02
Band gap	0.376 ± 0.01	0.26 ± 0.03	0.51 ± 0.03	0.87 ± 0.004
H_2 capacity	1.537 ± 0.06	4.31 ± 0.06	2.077 ± 0.01	0.98 ± 0.00
Xe uptake at HP	0.561 ± 0.002	0.582 ± 0.001	0.76 ± 0.00	0.95 ± 0.00
Pore diameter	1.08 ± 0.05	3.49 ± 0.16	1.86 ± 0.04	0.82 ± 0.01
ASA	360.2 ± 10.4	263866 ± 8837	513.61 ± 8.56	0.77 ± 0.0005
Density	0.13 ± 0.004	0.04 ± 0.002	0.19 ± 0.005	0.92 ± 0.01
CH_4 DC	24.4 ± 0.59	1062 ± 68.8	32.6 ± 0.01	0.78 ± 0.009

Table S15: The regression results for our model (powder x-ray diffraction (PXRD) & Precursors) is compared to a descriptor-based machine learning (ML) model (i.e. Descriptors ML),[3] a transformer-based model accepting MOFids (MOFormer)[9,10] and a crystal graph convolutional neural network (CGCNN, accepting 3D structures)[11] across various geometric (CH_4 deliverable capacity - DC, density, Xe uptake at HP - high pressure, CH_4 uptake at HP, accessible surface area - ASA), chemistry-reliant (CO_2 uptake at low pressure - LP) and quantum-chemical (band gap) properties. Furthermore, an ablation study was done showcasing the impact of multimodality across these properties (i.e. comparing our model - PXRD & Precursors to Precursors only and PXRD only). The results are reported in the average Spearman's Rank Correlation Coefficient (SRCC) and mean absolute error (MAE) done over n = 3 trials (please see the SI for the statistics). The results shown here are on CoRE-2019[1,3] (CO_2 uptake at LP, CH_4 uptake at HP, ASA, density, CH_4 DC), QMOF[6,14] (band gap) and hMOF[3,7] (Xe uptake at HP) datasets. Source data are provided as a Source Data file.

**Property**	**PXRD & Precursors**		**Precursors only**		**PXRD only**		**Descriptors ML**		**CGCNN**		**MOFormer**	
	**SRCC**	**MAE**	**SRCC**	**MAE**	**SRCC**	**MAE**	**SRCC**	**MAE**	**SRCC**	**MAE**	**SRCC**	**MAE**
CH_4 upt. HP	0.90	1.35	0.60	2.53	0.83	1.72	0.98	0.45	0.82	1.52	0.63	2.47
CO_2 upt. LP	0.70	0.84	0.53	1.00	0.48	0.01	0.83	0.54	0.77	0.73	0.54	0.95
Band gap	0.87	0.37	0.84	0.41	0.36	0.03	0.84	0.41	0.88	0.34	0.87	0.36
CH_4DC	0.79	24.4	0.47	38.4	0.72	28.5	0.94	10.6	0.77	25.6	0.50	36.5
Xe upt. HP	0.95	0.56	0.67	1.44	0.90	1.09	0.96	0.45	0.83	1.02	0.84	0.92
ASA	0.77	360	0.41	604	0.72	436	-	-	0.74	410	0.43	574
Density	0.92	0.13	0.70	0.23	0.87	0.01	-	-	0.91	0.12	0.73	0.22

6 Recommendation system details

6.1 Threshold details

For the recommendation system, a multi-output version of the XRayPro model was constructed such that it returns six different labels that are relevant to the applications explored (methane storage, hydrogen storage, xenon storage, DAC, carbon capture, band gap). If the relevant label exceeds some threshold, then it can be flagged as a "worth trying" or "very interesting" MOF. A summary of the thresholds for each application can be found in Table S16.

Table S16: Summary of thresholds used for each application in the recommendation system. It should be noted that for some metal-organic framework (MOF) thresholds, the top N percent of the distribution of data we have were used to define those. For example, for methane storage, the "very interesting MOF" threshold was taken from literature, whereas the "worth trying MOF" threshold is the top 25 percent of CoRE-MOFs[1,3] for methane uptake. Source data are provided as a Source Data file.

**Application**	Property of Interest	"Worth Trying" MOF	"Very Interesting" MOF
**Methane storage**[16,17]	Methane uptake at 65 bar (mol/kg)	Greater than 7.93 mol/kg	Greater than 12 mol/kg
**Hydrogen storage**[18]	Hydrogen storage capacity (g/L)	Greater than 27 g/L	Greater than 33.5 g/L
**Xenon storage**	Xenon uptake at 10 bar (mol/kg)	Greater than 6 mol/kg	Greater than 8 mol/kg
**Carbon capture**[19]	Carbon dioxide uptake at 0.15 bar (mol/kg)	Greater than 2 mol/kg	Greater than 3.6 mol/kg
**Direct-air capture (DAC)**	Log(KH)_CO_2	Greater than -3.69	Greater than -2.2
**Band gap**[20]	Band gap (eV)	1 to 3 eV	Less than 1 eV

6.1.1 Justifications for thresholds

1. **Methane storage**: Anything above 12 mol/kg is considered very promising, as that is approximately the uptake at 65 bar for HKUST-1, which is a commonly used MOF in gas storage applications.[16,17] 7.93 mol/kg was chosen, as that was the cutoff to retrieve

the top 30 percent of MOFs (in terms of methane uptake at 65 bar) available in the used CoRE-MOF database;[1,3]

2. **Hydrogen storage**: For balanced hydrogen capacity, IRMOF-20 is known to be a "record-holder", with an available capacity of 33.4 g H_2/L - hence why this criteria was used for flagging very promising MOFs for hydrogen capture.[18] 27 g/L was chosen, as it was the cutoff to retrieve the top 30 percent of MOFs (in terms of hydrogen capacity) available in the used hMOF database;[7]

3. **Xenon storage**: Due to the lack of experimental data online for xenon storage in MOFs, the top 10 and 30 percent of xenon uptakes in the hMOF database were chosen to be the thresholds to flag a MOF as "very promising" and "promising" respectively;[7]

4. **Carbon capture**: Mahajan et al. (2022) stated that for a promising candidate for carbon capture, anything greater than 2 mmol CO_2/g adsorbent is acceptable.[19] 3.6 mol/kg was calculated from retrieving the top 10 percent of MOFs from the available CoRE-MOF database;[1,3]

5. **Direct-air capture**: Due to the lack of available experimental data online, the top 10 and 30 percent of the Henry coefficient of solubility for CO_2 were taken to be the thresholds for a very promising and promising MOF respectively;[1,3]

6. **Band gap**: Zhang et al. (2024)[20] stated that MOFs with band gap between 1-3 eV exhibit semiconductor behaviour and are appropriate to use as sensors, microelectronics, etc.. Anything below this range i.e. 1 eV is chosen to be a very promising MOF, band gap wise.[6,14]

6.2 Masked loss function used

One challenge that was faced is how many rows in the concatenated dataframe across multiple datasets (CoRE-2019,[1,3] BW20K,[4] ARABG, hMOF,[7] QMOF[6,14]) had unlabelled data. For

example, while CoRE,[1,3] BW20K,[3,4] ARABG[3] contained data on methane uptake at 65 bar, it lacked band gap data. Rather than doing direct supervised learning, we utilized masked loss functions that ignored entries in which there was no data. The mask can be defined as:

$$M_i = \begin{cases} 1 & \text{if isfinite}(y_{\text{true},i}) \\ 0 & \text{otherwise} \end{cases} \tag{1}$$

The masked loss function L_{masked} for some arbitrary regression loss function can then be written as:

$$L_{\text{masked}} = \frac{\sum_{i=1}^{n} M_i \cdot L(y_{\text{true},i}, y_{\text{pred},i})}{\sum_{i=1}^{n} M_i} \tag{2}$$

7 Statistics of recommendations system

Please refer to Table S16 on a summary on what makes a metal-organic framework (MOF) interesting/not interesting for a particular application for these following sections.

7.1 Recommendations on computation-ready MOFs

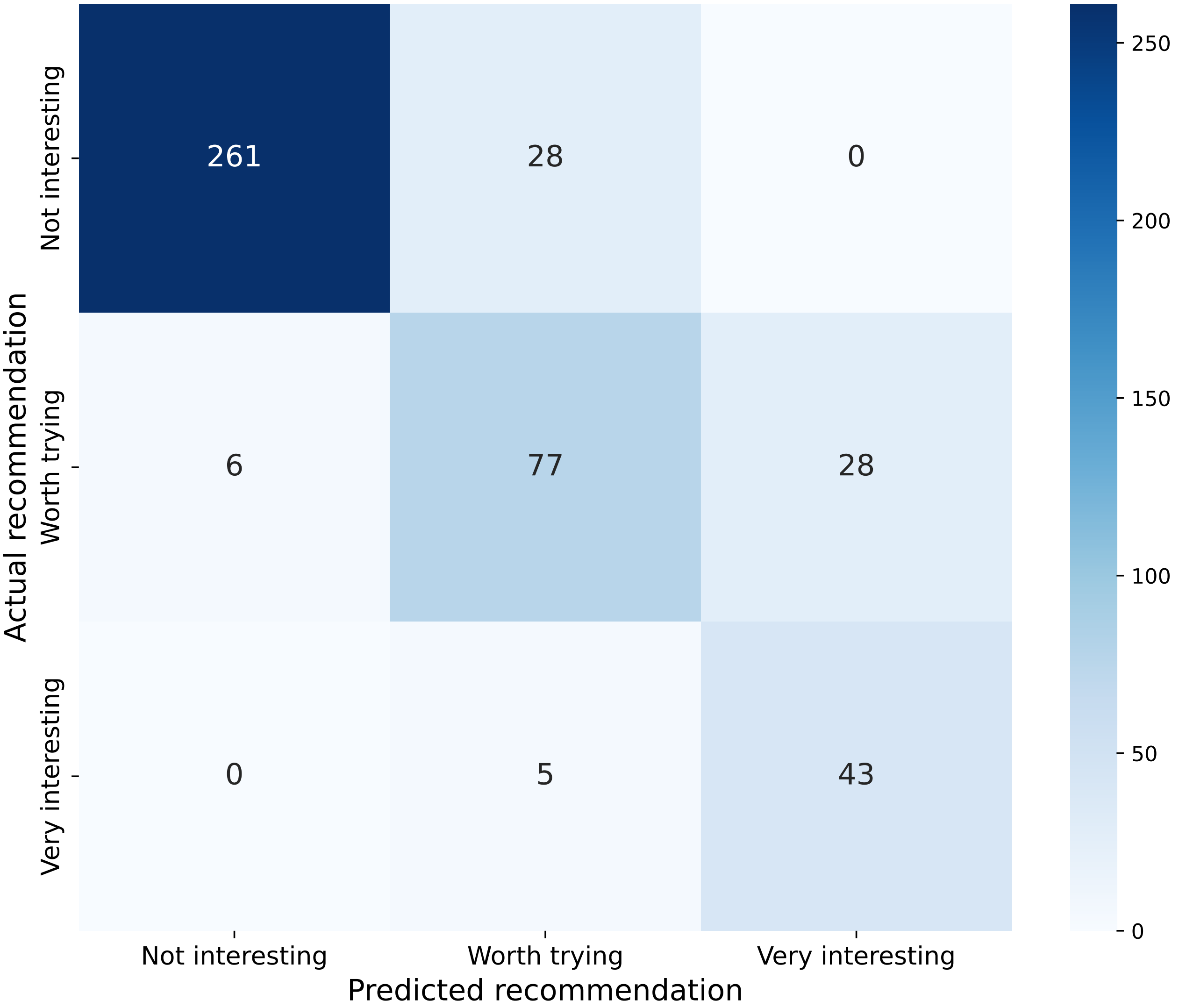

Figure S. 7| Confusion matrix for H_2 storage from our metal-organic framework (MOF) recommendation system. The overall F1-score is reported as 0.856, with recall being 0.850. Source data are provided as a Source Data file.

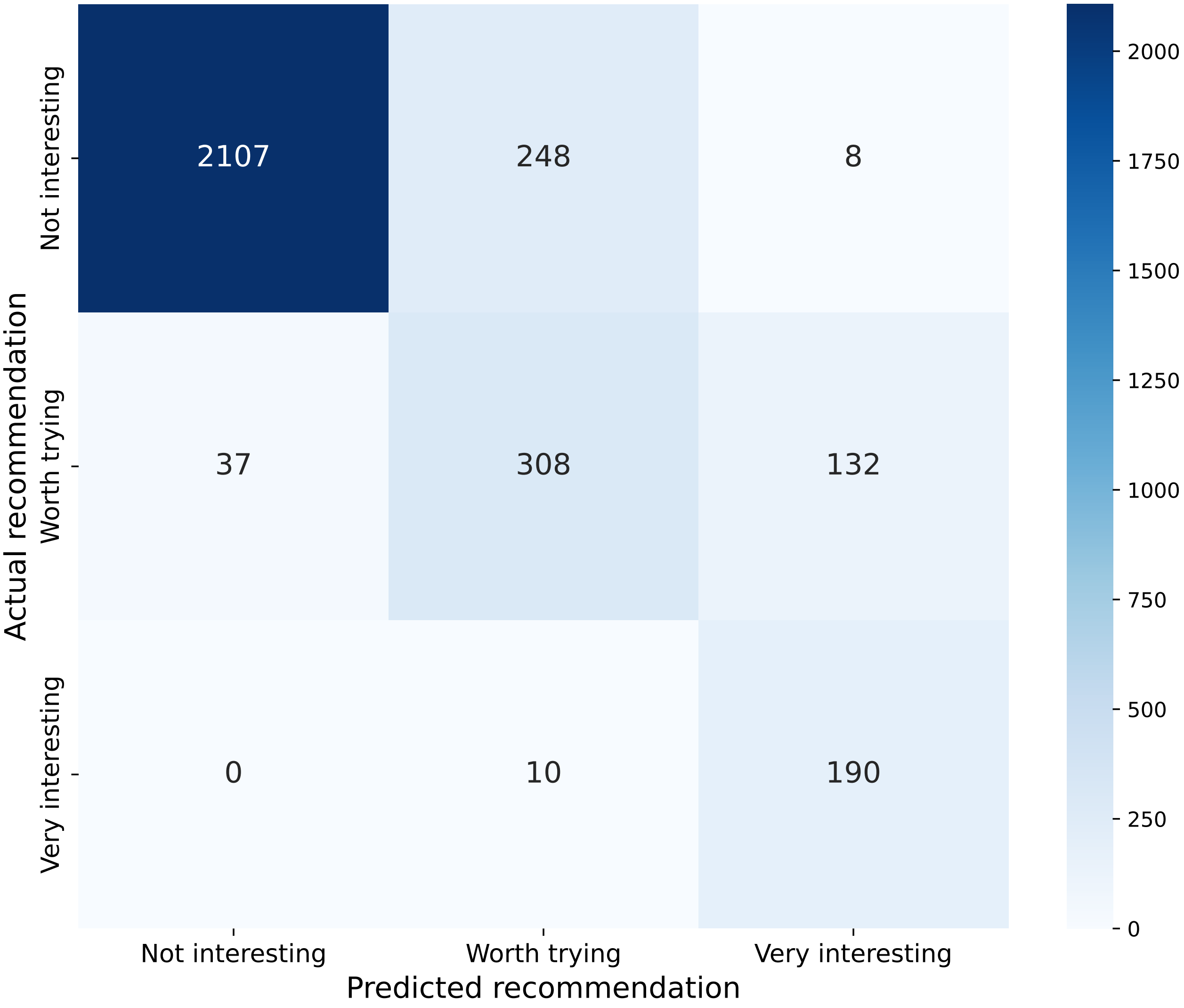

Figure S. 8| Confusion matrix for Xe storage from our metal-organic framework (MOF) recommendation system. The overall F1-score is reported as 0.867, with recall being 0.857. Source data are provided as a Source Data file.

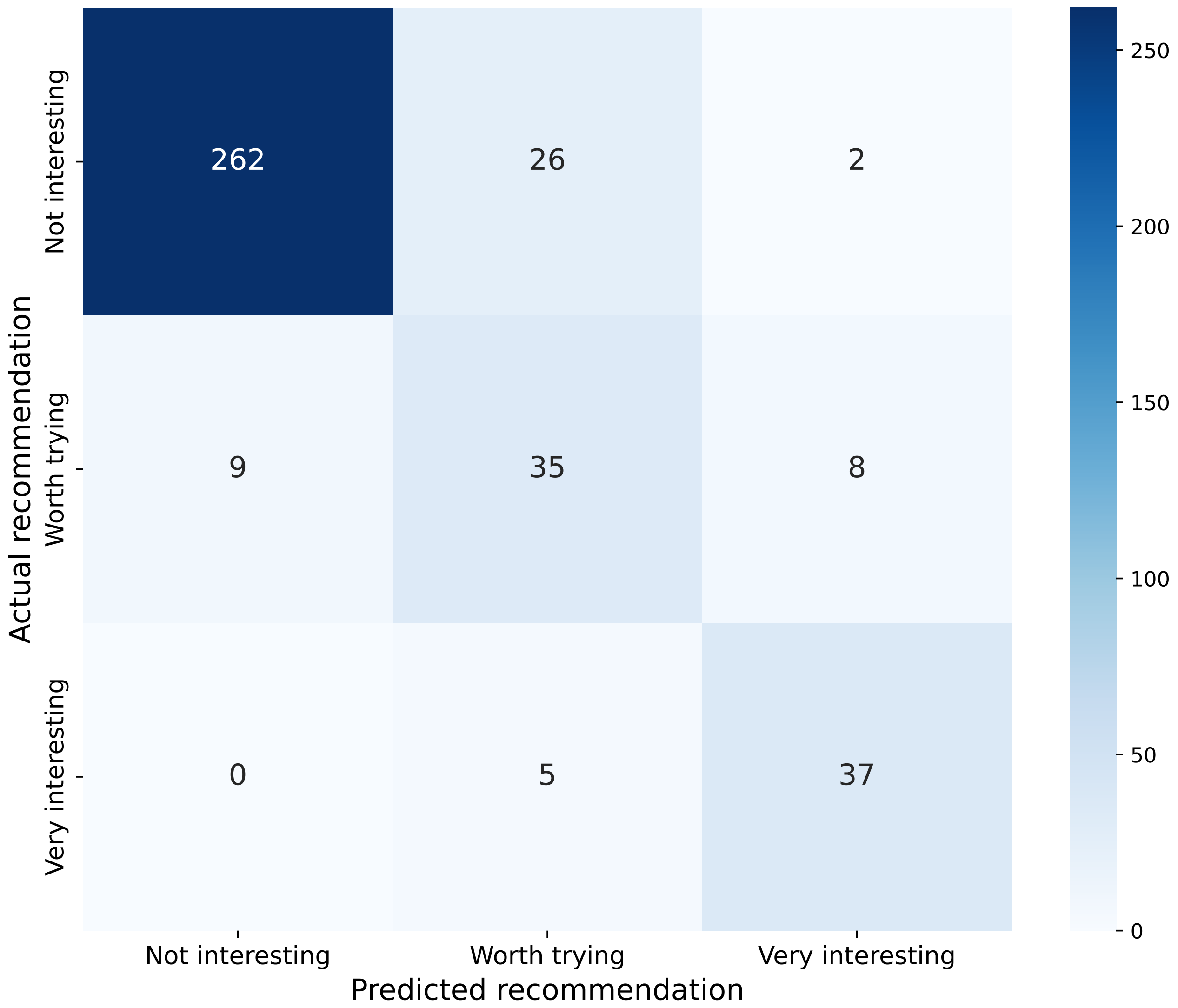

Figure S. 9| Confusion matrix for CH_4 storage from our metal-organic framework (MOF) recommendation system. The overall F1-score is reported as 0.877, with recall being 0.870. Source data are provided as a Source Data file.

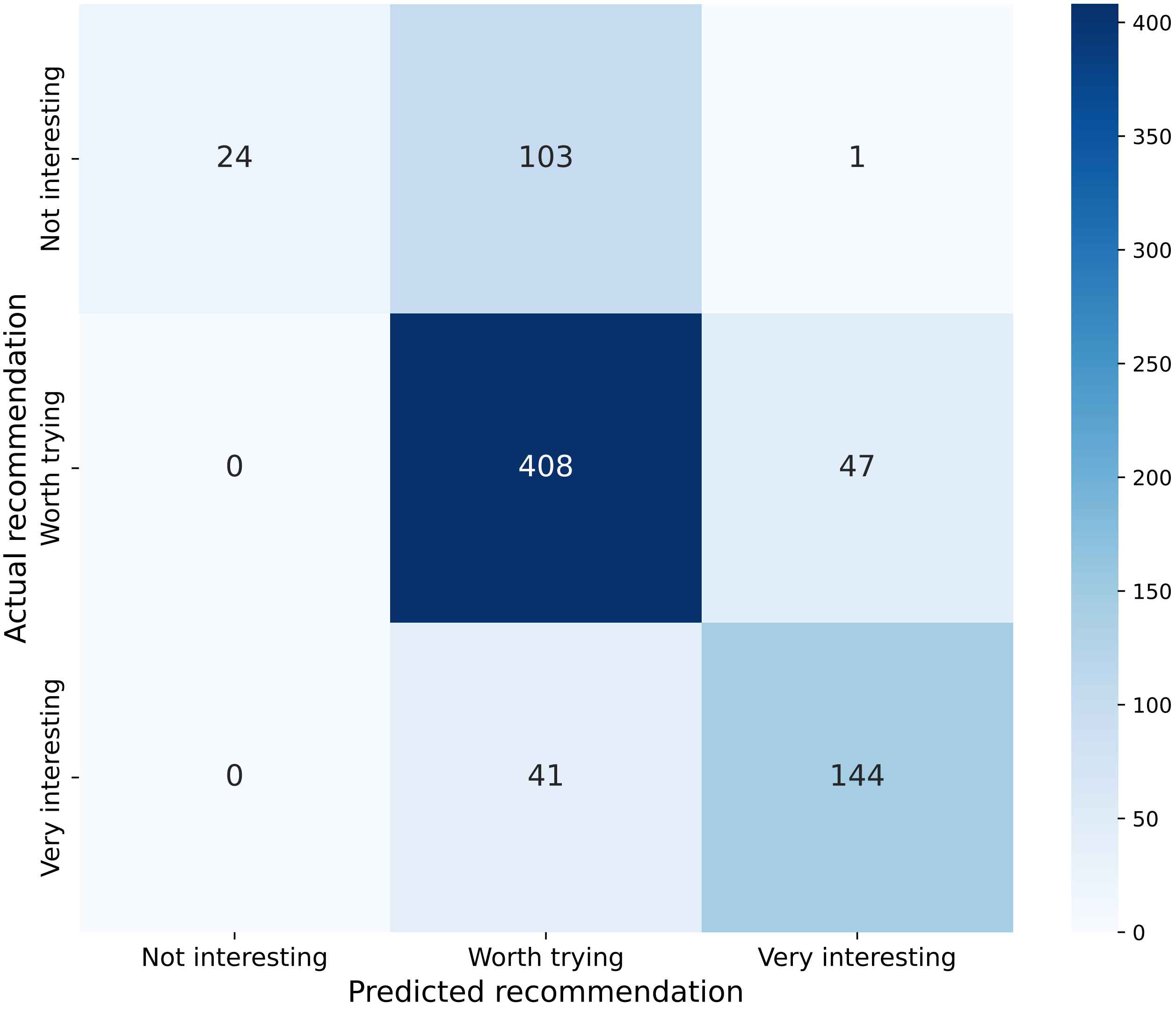

Figure S. 10| Confusion matrix for band gap from our metal-organic framework (MOF) recommendation system. The overall F1-score is reported as 0.717, with recall being 0.750. Source data are provided as a Source Data file.

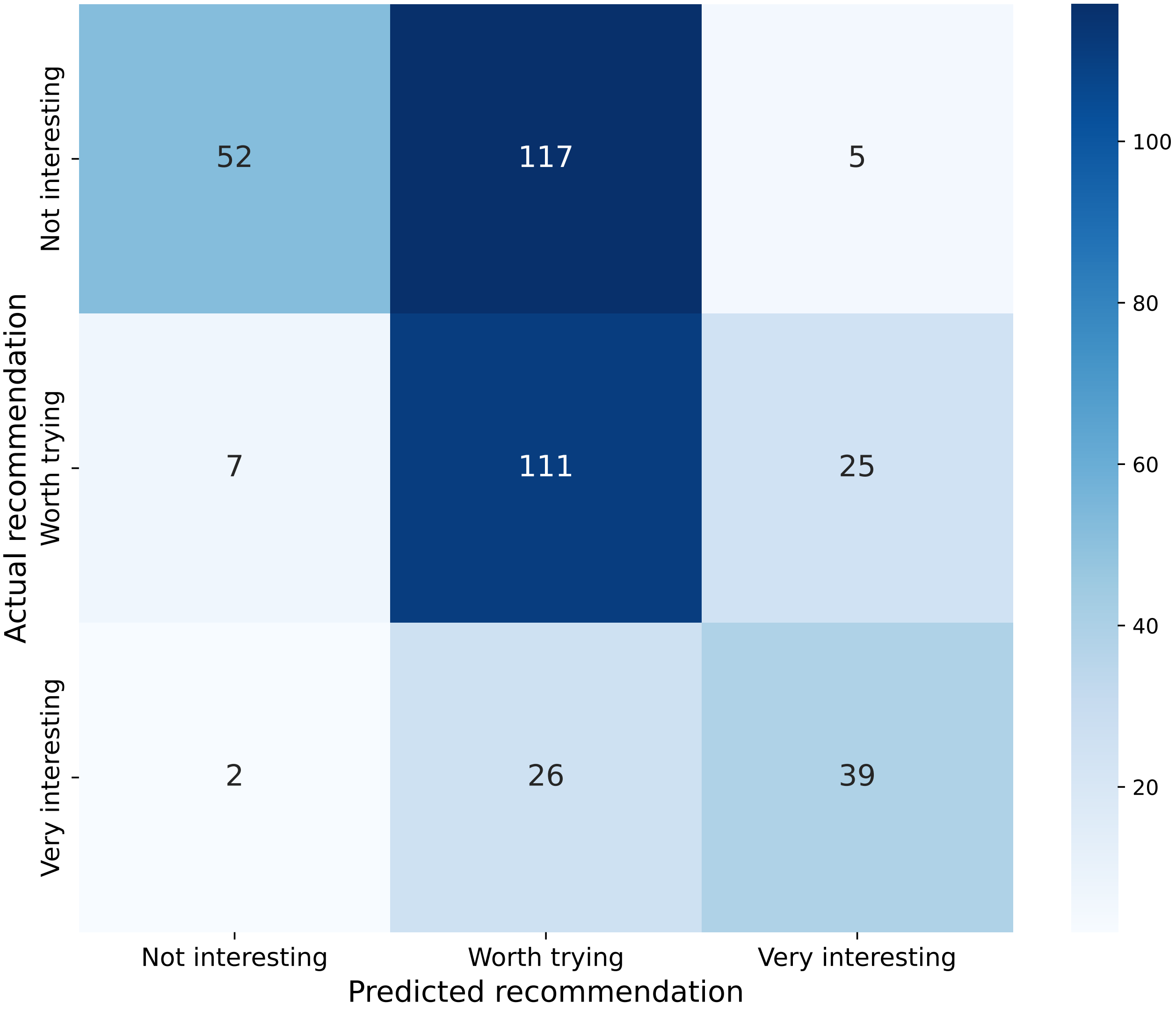

Figure S. 11| Confusion matrix for direct-air capture (DAC) from our metal-organic framework (MOF) recommendation system. The overall F1-score is reported as 0.509, with recall being 0.526. Source data are provided as a Source Data file.

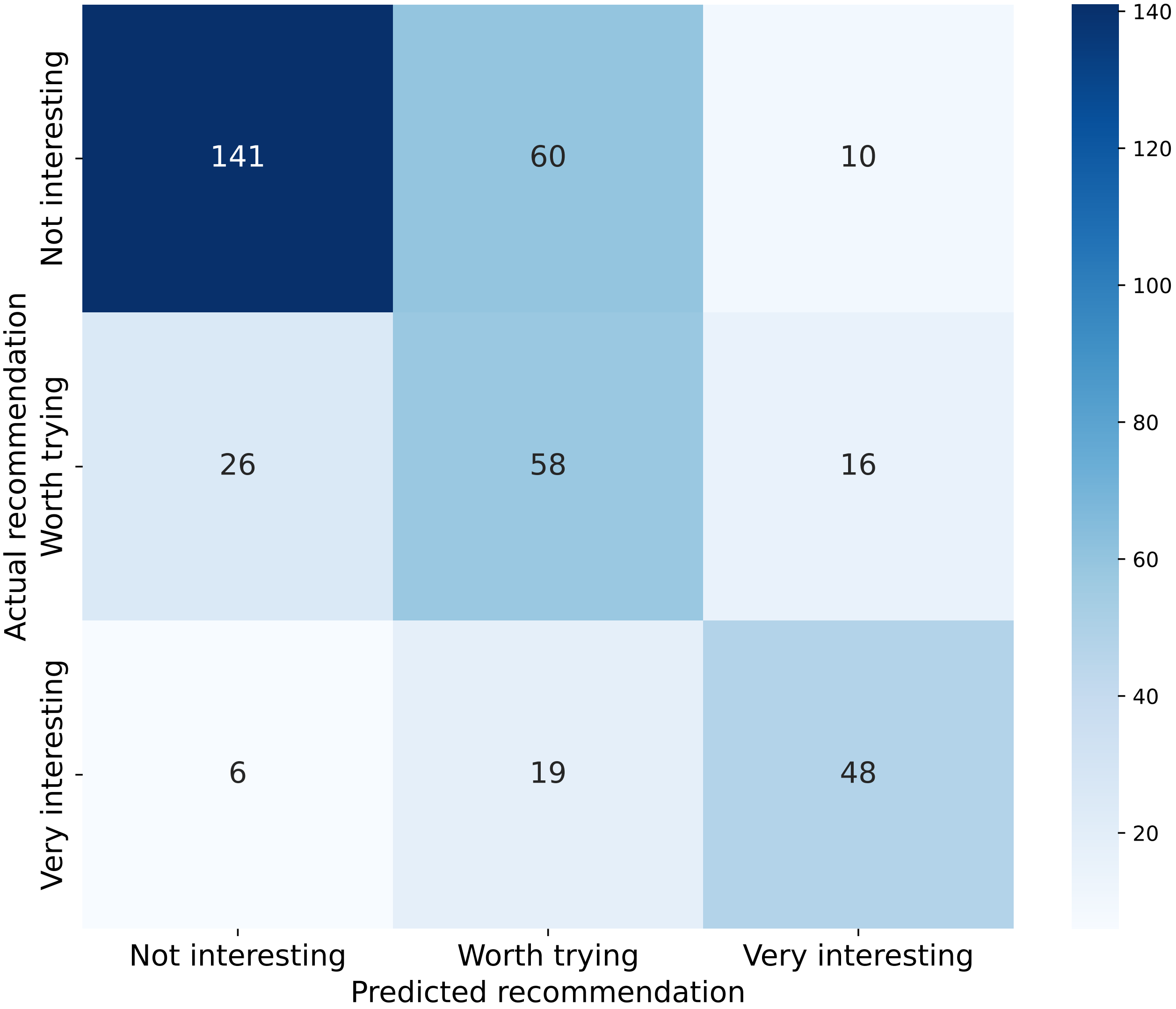

Figure S.12| Confusion matrix for carbon capture from our metal-organic framework (MOF) recommendation system. The overall F1-score is reported as 0.655, with recall being 0.643. Source data are provided as a Source Data file.

7.2 Recommendations on Cambridge Structural Database (CSD) MOFs

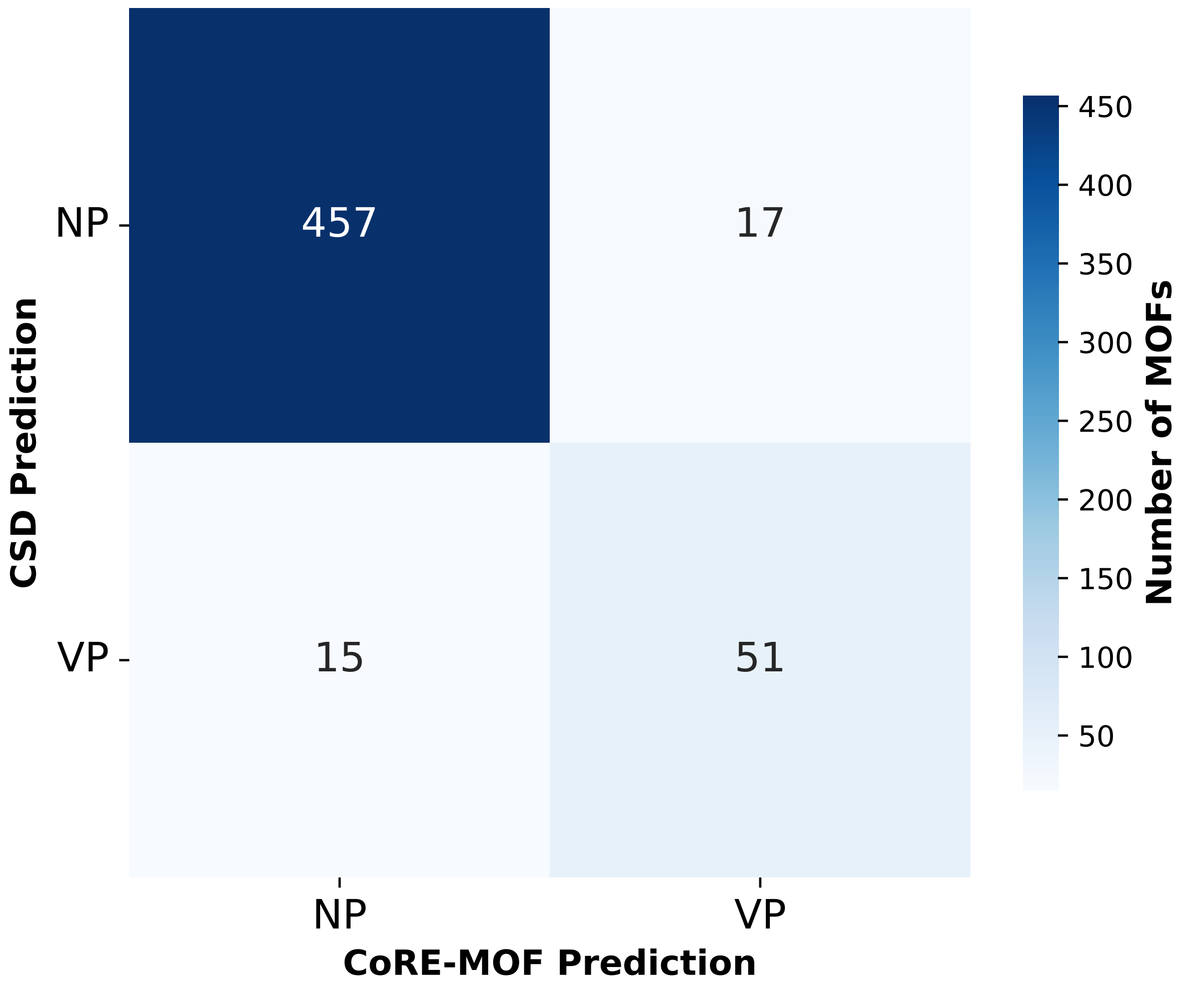

Figure S. 13| Confusion matrix for predictions on methane storage recommendation when using Cambridge Structural Database (CSD)[21] powder x-ray diffraction (PXRD) patterns versus CoRE-MOF PXRDs.[1,3] This is for binary classification i.e. not promising (NP/P, anything below 12 mol/kg) and VP (anything above 12 mol/kg). The F1, recall and precision are 0.76, 0.75 and 0.78 respectively. Please refer to Table S16 for details on the selected thresholds for this evaluation. Source data are provided as a Source Data file.

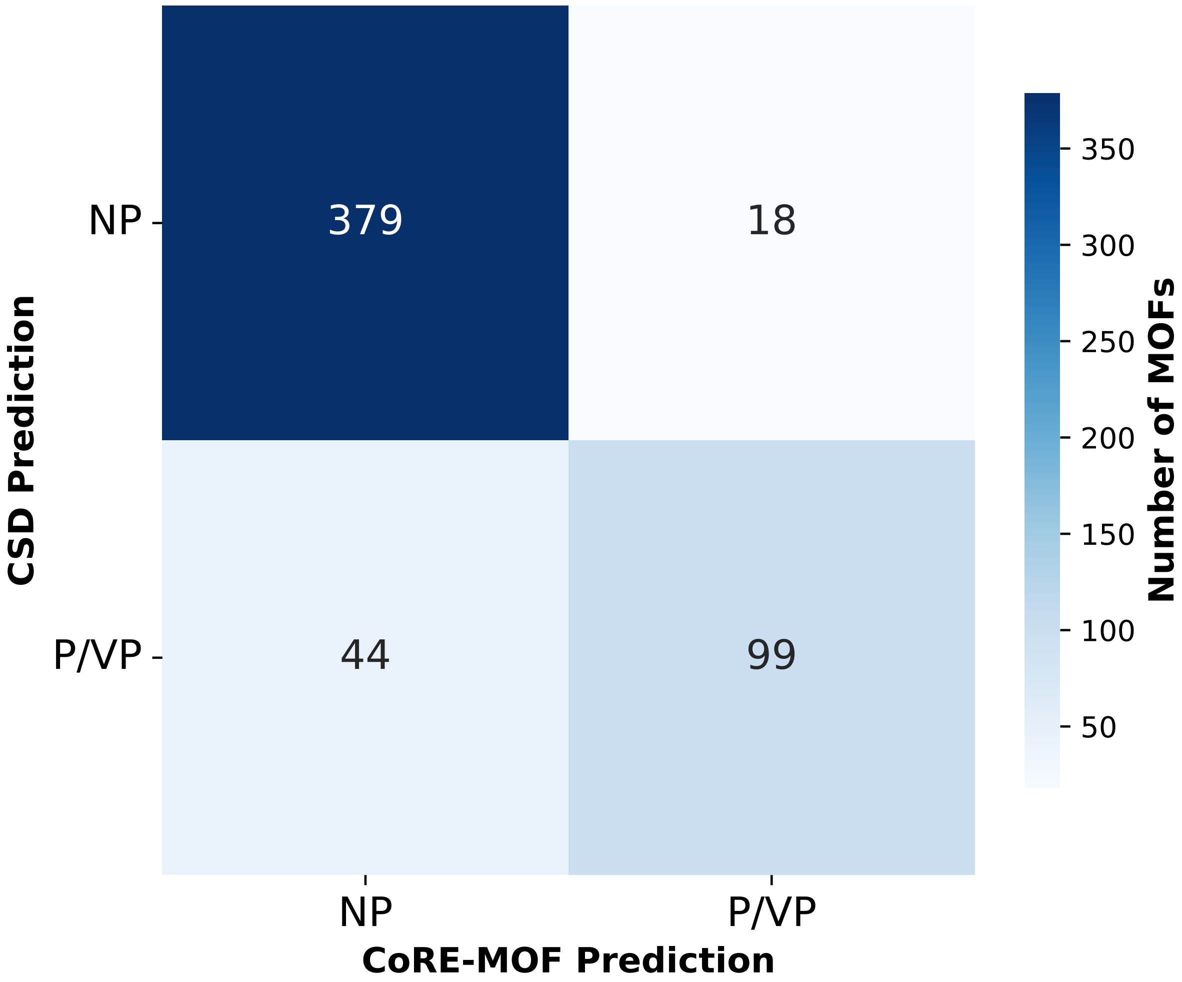

Figure S. 14| Confusion matrix for predictions on methane storage recommendation when using Cambridge Structural Database (CSD)[21] powder x-ray diffraction (PXRD) patterns versus CoRE-MOF PXRDs.[1,3] This is for classifying MOFs that are either not promising for methane storage (below 7.93 mol/kg), or promising/very promising (greater than or equal to 7.93 mol/kg). The F1, recall and precision are 0.76, 0.69 and 0.85 respectively. Please refer to Table S16 for details on the selected thresholds for this evaluation. Source data are provided as a Source Data file.

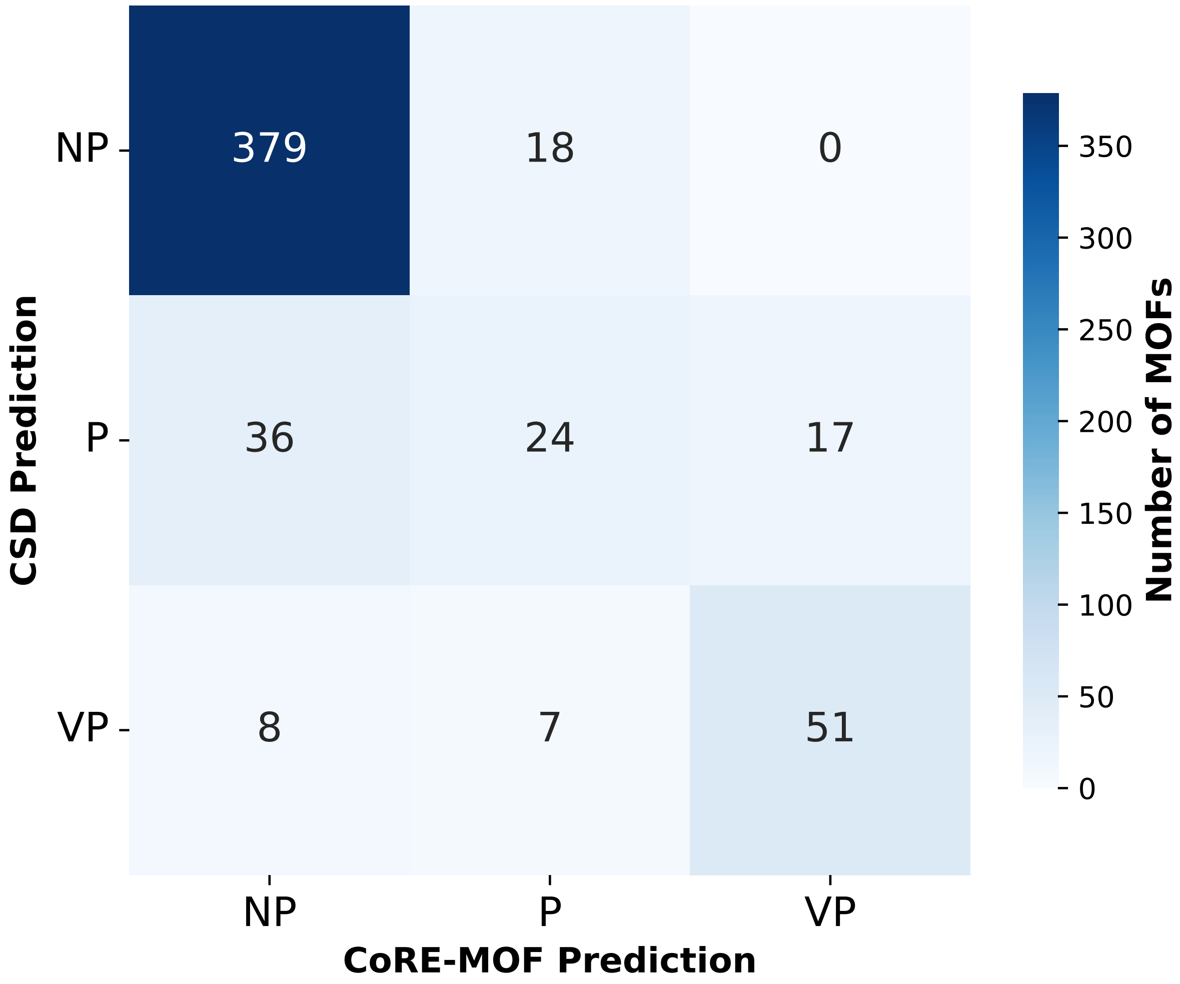

Figure S. 15| Confusion matrix for predictions on methane storage recommendation when using Cambridge Structural Database (CSD) powder x-ray diffraction (PXRD) patterns versus CoRE-MOF PXRDs.[1,3] This is for tertiary classification i.e. not promising (NP, anything below 7.93 mol/kg), promising (anything between 7.93 mol/kg and 12 mol/kg) and VP (anything above 12 mol/kg). The F1, recall and precision are 0.83, 0.84 and 0.82 respectively. Please refer to Table S16 for details on the selected thresholds for this evaluation. Source data are provided as a Source Data file.

8 "Time travel" model for MOF application discovery

The time travel model was constructed to showcase the model's ability to find new applications for MOFs that are in the future. From using the CCDC API, it is possible to get the year a MOF was deposited into the database. Afterwards, the MOFs that were deposited from the year 2017 onwards were put aside into a test set (size: 160 entries), and the rest of the MOFs were used as the train set (size: 3,485 entries). The label chosen was carbon dioxide at 0.15 bar, as then we can see if the model is capable of finding carbon capture as a new application for some MOFs. The model was then trained for 100 epochs, giving an SRCC score of 0.72 and MAE of 0.89 mol/kg, which is around the same as when the pre-trained model was finetuned without the year constraint. Table S17 shows all the promising MOFs flagged by our model, showcasing the correctly flagged MOFs (within 15 percent of the threshold), incorrectly flagged MOFs and the MOFs already synthesized for carbon capture. This showcases that the model is capable of recommending MOFs that will be synthesized for future use by training it on pre-existing MOFs.

Table S17: Predicted promising metal-organic frameworks (MOFs) for carbon capture using the "time-travel" XRayPro model (i.e. our model) with their corresponding intended applications. The rows highlighted in green are MOFs correctly flagged by the system (within 15 percent), blue rows indicate that the MOF was intended to be synthesized for carbon capture (flagged correctly, but not a new application discovered) and the red rows indicate MOFs incorrectly flagged. The MOFs detected include EXUHUC,[22] NAMDUD,[23] NAWKII,[24] OZAVES,[25] GARLUJ,[26] OZAVUI,[27] GARLIX,[26] DACYUE,[28] LAQZOV,[29] INOMOP,[30] TAQGUQ,[31] LARNEA,[32] KAPHUH,[33] DACZUF,[34] IYAZAL,[35] GARLET,[26] GARLOD[26] and CAPLOX.[36] Source data are provided as a Source Data file.

Predicted Promising MOFs	FF CO_2 uptake at 0.15 bar (mol/kg)	Intended Application
EXUHUC	4.054856	Thermal stability
NAMDUD	3.688322	Gas adsorption of CO_2 and separation
NAWKII	6.296414	Catalyst
OZAVES	4.563314	Photoluminescence
GARLUJ	3.533759	Some luminescence application
OZAVUI	3.974213	Photoluminescence
GARLIX	3.590423	Some luminescence application
DACYUE	4.985886	High performing catalyst
LAQZOV	5.705628	Adsorption/removal of fluoride from water
INOMOP01	5.532464	Photoluminescence
TAQGUQ	1.742203	Stability in humid conditions
LARNEA	0.960394	Thermal stability
KAPHUH	3.227573	C_2H_2/C_2H_4 separation
DACZUF	4.581936	Photoluminescence and thermal stability
IYAZAL	4.543934	Luminescence and thermal stability
GARLET	3.619186	Luminescence
GARLOD	3.551558	Luminescence
CAPLOX	5.147092	Rapid sensing of copper ions

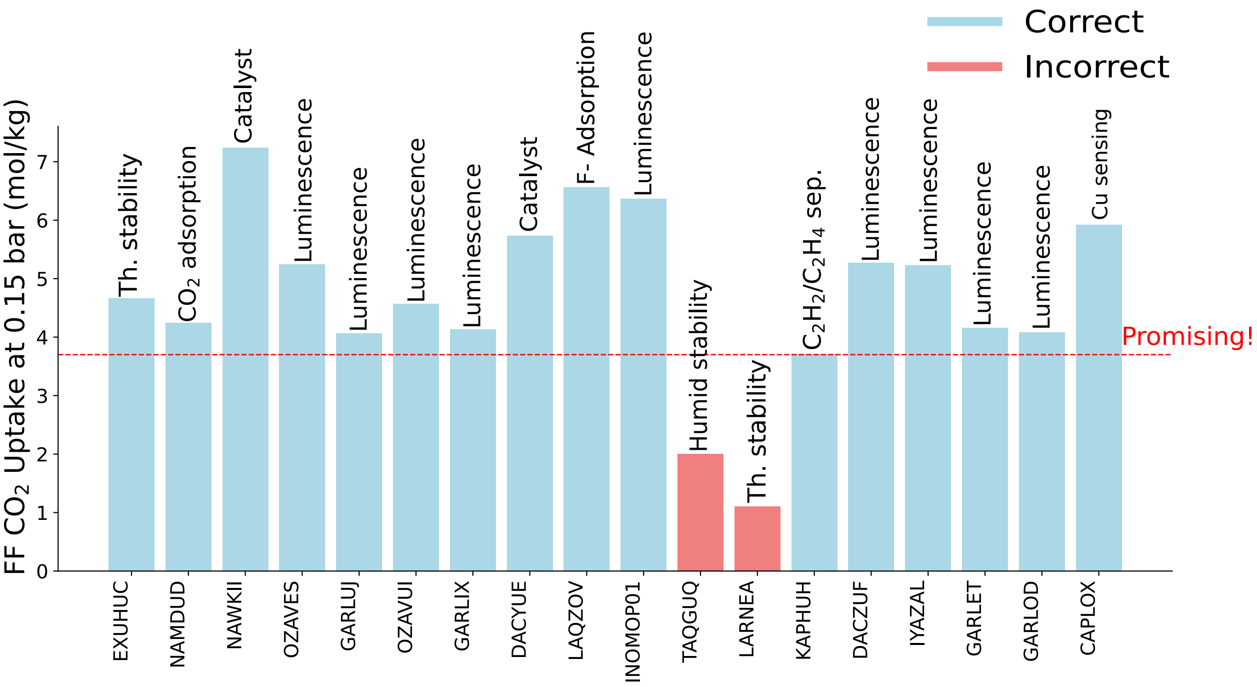

Figure S. 16| Results for time-travel model, with an expansion made to the rest of the metal-organic frameworks (MOFs) (by their Cambridge Structural Database - CSD- reference codes) that were flagged by our system, while comparing it with the MD-calculated uptake labels at LP (low pressure). It should be noted that "Th." is short for "thermal" in this figure (i.e. Th. stability: thermal stability). The MOFs detected include EXUHUC,[22] NAMDUD,[23] NAWKII,[24] OZAVES,[25] GARLUJ,[26] OZAVUI,[27] GARLIX,[26] DACYUE,[28] LAQZOV,[29] INOMOP,[30] TAQGUQ,[31] LARNEA,[32] KAPHUH,[33] DACZUF,[34] IYAZAL,[35] GARLET,[26] GARLOD[26] and CAPLOX.[36] Source data are provided as a Source Data file.

9 Similarity Analysis of Time-Travel Sets

A concern when assessing the viability of our time-travel experiment was the concern of visible similarity between the train set (i.e. MOF entries deposited before 2017) and the test set (MOF entries deposited after and including 2017). If the sets were visibly similar, it would imply that the experiment done would be flawed as the model would have memorized the patterns seen in the training set embedding space. To make a fair comparison, a baseline similarity matrix was computed by training a model on carbon dioxide uptake at low pressure (appropriate for carbon capture) from performing a random train/test split, retrieving the embeddings of the sets and computing the cosine similarity matrix between Z_{train} and Z_{test}:

$$S_c = \frac{Z_{\text{train}} \cdot Z_{\text{test}}}{\|Z_{\text{train}}\| \, \|Z_{\text{test}}\|} \tag{3}$$

From this, from using the same methodology, the cosine similarity matrix between the train and test embeddings from the time-travel experiment were computed. Both the baseline random splitting and time-travel sets achieve a low (and more importantly, similar) mean cosine similarity of around 0.35, showing that the split is no less arbitrary than random splitting, while retaining its low train/test similarity. Figure S 17 showcases the baseline (panel (a)) and time-travel sets (panel (b)).

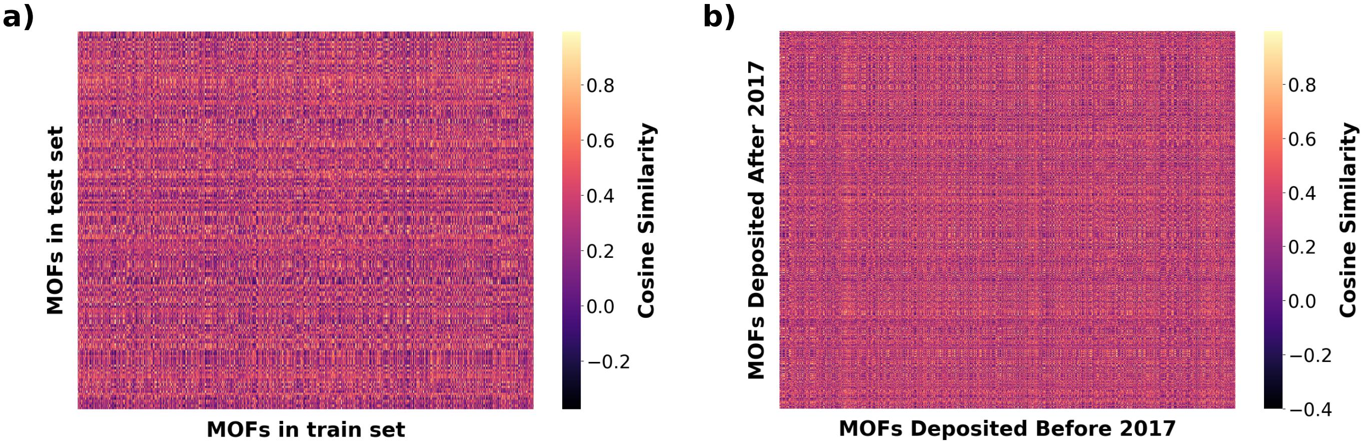

Figure S.17| Similarity analysis between model embeddings for train and test sets for "time-travel" model For a baseline, a simple random train/test split was done and used to train a model on predicting the CO_2 uptake at low pressure for metal-organic frameworks (MOFs) from the CoRE-2019 database,[1,3] with the train and test model embedding cosine similarities computed in panel (a). For panel (b), a similar computation was done on the train (i.e. MOFs deposited into the Cambridge Structural Database, CSD,[21] before 2017) and test (MOFs deposited after and including 2017) sets for the "time-travel" model. Both splits are shown to have low mean cosine similarity (around 0.35 for both). Source data are provided as a Source Data file.

10 Evaluation of CoRE-MOF 2019's diversity

While chemistry-reliant properties are understandably tricky to predict with PXRD patterns alone due to the PXRD pattern mainly providing information about the crystal structure and geometry, the geometry predictions for CoRE-MOF 2019 were tricky to predict in comparison to the other databases (BW20K, ARABG, COF, hMOF) shown. As a result, a couple of assessments were done to determine why this was the case. Initially, the diversity of the topology was thought to be the reason, as CoRE has more than 350 unique topologies. However, the COF database is easy for the model to predict despite having 342 unique topologies. From past works, it was shown that rather than topologies, less symmetric crystal systems are harder to predict.[37] Thus, the crystal systems were compared between CoRE and a database the model performed well on (BW20K), and the model performance

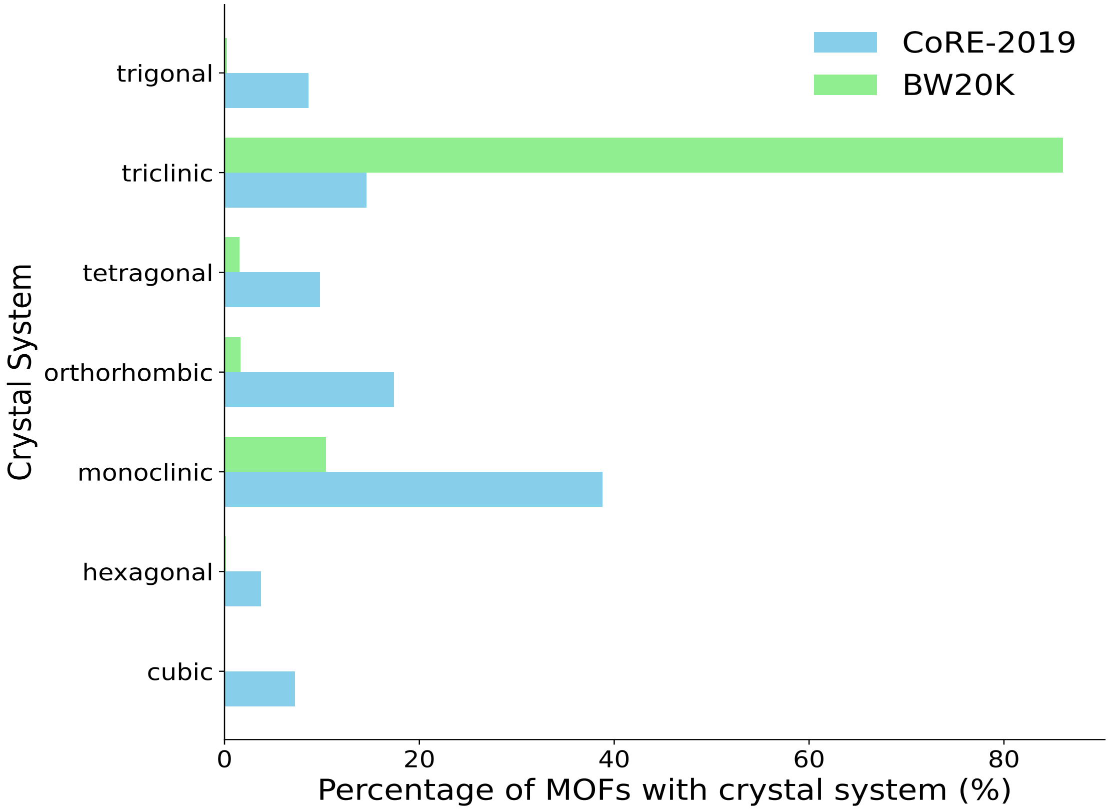

Figure S. 18| Comparison between the CoRE-2019[1] crystal systems vs. BW20K[4] crystal systems. This showcases a couple of noteworthy points: there is a more even distribution of crystal systems in CoRE-2019 whereas BW20K is heavily biased towards triclinic and monoclinic crystal systems. While it seems like there are no trigonal, hexagonal and cubic entries in BW20K from the figure, it should be noted that they are present - there are just significantly limited number of entries with these crystal systems. This is important when commenting on the difference in results for the geometric property predictions between the two datasets. Source data are provided as a Source Data file.

between crystal systems was evaluated. The model used to assess this was Suzuki et al. (2020)'s extremely randomized trees model.[37]

For BW20K, it can be seen that there is a heavy bias towards triclinic MOFs and very little MOFs with high symmetry. As a result, there was an expectation for the model to be heavily biased towards performing well on the less symmetric MOFs (triclinic, monoclinic, orthorhombic) as most of the train set (when randomly split) is going to be mostly less symmetric MOFs. However, for the more symmetric MOFs, there is an expectation for the

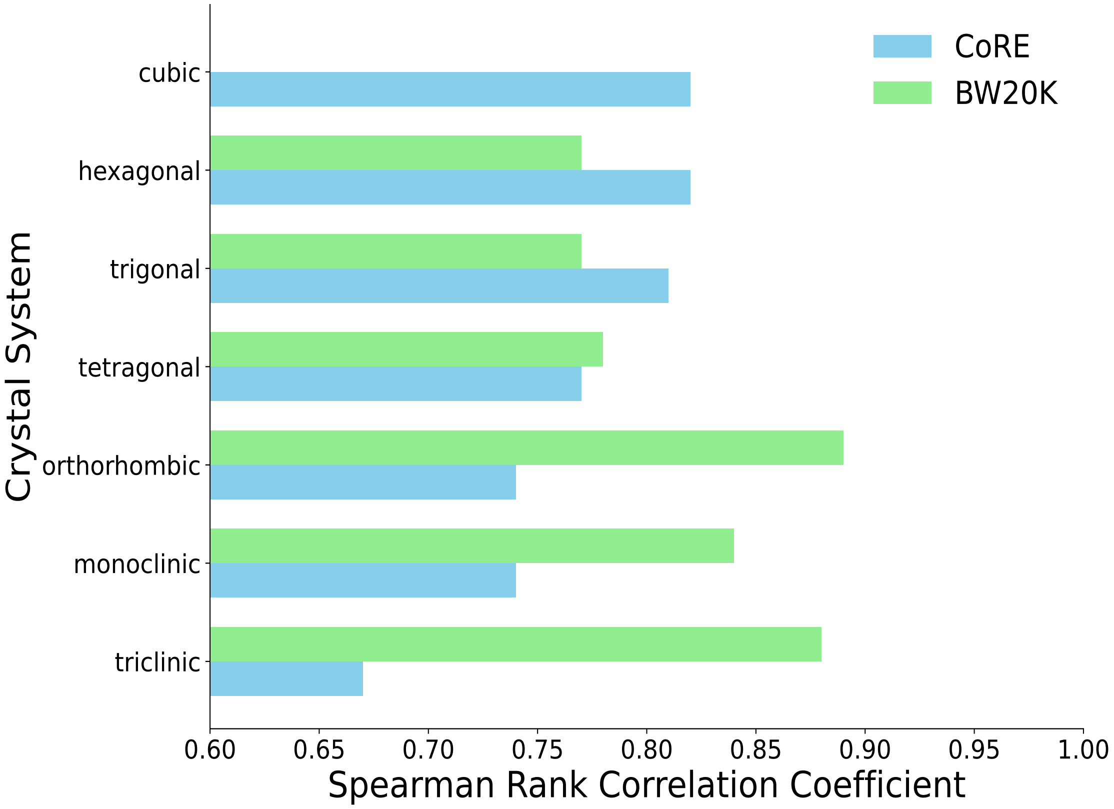

Figure S. 19| Evaluation of Suzuki et al. (2020)'s model[37] and comparing the performance between CoRE-2019[1] and BW20K[4] depending on the crystal system. This showcases that BW20K model performance is good due to most of the crystal system examples in the database being triclinic, as the SRCC for triclinic is around 0.88, whereas the more symmetric crystal systems are harder for it to predict. When CoRE is used, it can be seen that the more symmetric MOFs are easier to predict due to the more diverse distribution in crystal systems, and this may cause a bias in the model that makes it progressively harder to predict less symmetric MOFs such as triclinic or monoclinic systems. Source data are provided as a Source Data file.

model trained on BW20K to struggle in predictions - which is exactly the case.

However, there is a more even distribution in crystal systems for CoRE (Figure S18). From the results in Figure S19, while the more symmetric MOFs give healthy model performance, the less symmetric MOFs are more difficult to predict - and as a good proportion of the CoRE dataset is triclinic, monoclinic and orthorhombic, the model performance is going to be poor as these are not symmetrical MOFs.

The reason as to why this is the case can be found in a case study with the results in Figure S20. In this study, we took the CoRE database and created train sets only having one crystal system and tested on only one crystal system as well. For instance, the first row of the heat map is where the train set only consisted of triclinic MOFs, and for each column, it was tested on only triclinic MOFs, only monoclinic MOFs, etc. It can be seen that when triclinic MOFs (the least symmetric system) are used as the train set, the results for the test set are quite consistent across all symmetries. However, when the model is trained on only cubic MOFs (the most symmetric system), the model performance is very poor on less symmetric MOFs such as triclinic, monoclinic and orthorhombic and increases as the test set's symmetry increases. This is important because it displays how the presence of more symmetric MOFs in the database skews the model performance away from predicting geometric properties well on less symmetric MOFs.

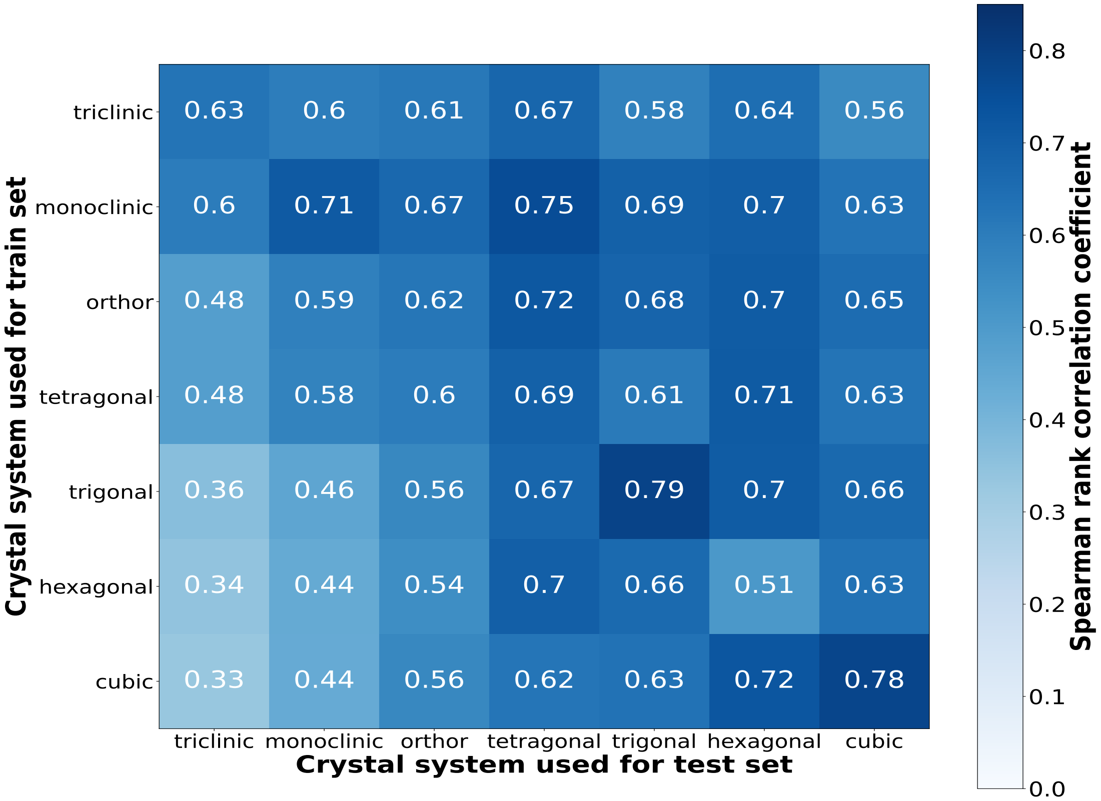

Figure S. 20| Evaluation of Suzuki et al. (2020)'s model[37] on CoRE-MOF 2019[1] database while using varying train and test set, depending on crystal system. These results indicate that when using only less symmetric metal-organic frameworks (MOFs) as train sets, it has consistent performances across all available symmetries for the test sets. However, when only very symmetric MOFs (such as cubic) are used as the train sets, when the test set only consists of less symmetric MOFs, the model performance is poor and the performance increases with increasing symmetry. Source data are provided as a Source Data file.

11 Model robustness on experimental PXRD patterns

The experimental PXRDs from Howarth et al. (2024) and Pougin et al. (2024)[38] were transformed using the procedure outlined in the Methodology section of the main print. The model outputs from each PXRD are given in Table S18. In this case, methane uptake at 65 bar was predicted because it is impacted the most by changes in PXRD due to it being a geometric property. The relative errors were calculated by the following expression:

$$\textit{Relative error} = \frac{|y_{\text{Sim}} - y_{\text{Exp}}|}{\mathbb{D}} \times 100\% \tag{4}$$

Where $\mathbb{D}$ is the domain of the data containing methane uptake at 65 bar labels. This data's outliers were removed, giving a minimum value of 0.18 mol/kg and maximum value of 19.65 mol/kg. Thus:

$$\textit{Relative error} = \frac{|y_{\text{Sim}} - y_{\text{Exp}}|}{y_{\text{max}} - y_{\text{min}}} \times 100\% \tag{5}$$

Using a similar approach for hydrogen storage capacity, the domain is around 37 g/L.

Table S18: Our model outputs for methane uptake at 65 bar (mol/kg)[3] predictions on metal-organic frameworks (MOFs) when a simulated powder x-ray diffraction (PXRD) pattern is inputted versus its corresponding experimental PXRD to prove model robustness and evaluate how the recommendation system assesses a promising MOF or not. N.I = Not interesting, W.T = Worth trying, V.I = Very interesting. Source data are provided as a Source Data file.

**MOF**	**Simulated output (mol/kg)**	**Experimental output (mol/kg)**	**Relative Error (%)**
**CAU-28**	4.32 (N.I)	3.31 (N.I)	5.31
**Yb-UiO-66**	6.43 (W.T)	9.35 (W.T)	7.39
**Zn2-(TBAPy) CW**	6.11 (N.I)	2.62 (N.I)	18.4
**Zn2-(TBAPy) dist**	4.62 (N.I)	3.68 (N.I)	4.96
**Zn-(Ade)(TBAPy) ions**	5.72 (N.I)	10.60 (V.I)	25.66
**Zn-(6BA)(TBAPy) CW**	5.28 (N.I)	13.40 (V.I)	42.71
**Zn-(6BA)(TBAPy) OMS**	2.98 (N.I)	20.98 (V.I)	94.74

Table S19: Our model outputs for hydrogen storage capacity with a pressure swing from 100 to 5 bar (g/L)[7] predictions on metal-organic frameworks (MOFs) when a simulated powder x-ray diffraction (PXRD) pattern is inputted versus its corresponding experimental PXRD to evaluate model robustness and the recommendation system's evaluation of promising MOFs. N.I = Not interesting, V.I = Very interesting. Source data are provided as a Source Data file.

**MOF**	**Simulated output (g/L)**	**Experimental output (g/L)**	**Relative Error (%)**
**CAU-28**	22.64 (N.I)	19.85 (N.I)	7.35
**Yb-UiO-66**	8.63 (N.I)	16.23 (N.I)	16.23
**Zn2-(TBAPy) CW**	3.63 (N.I)	1.53 (N.I)	5.53
**Zn2-(TBAPy) dist**	3.61 (N.I)	3.44 (N.I)	0.45
**Zn-(Ade)(TBAPy) ions**	10.12 (N.I)	11.93 (N.I)	4.73
**Zn-(6BA)(TBAPy) CW**	6.72 (N.I)	14.98 (N.I)	21.65
**Zn-(6BA)(TBAPy) OMS**	3.48 (N.I)	29.43 (V.I)	68.03

12 Evaluation of model on covalent-organic frameworks

The model was evaluated on covalent-organic frameworks (COFs) as a way to test its transferability to other materials.[39?] It should be noted that MOFids could not be computed for COFs, so only PXRD was used as the input into the model. The model was then evaluated on methane uptake at high pressure (v STP/v), crystal density (kg/m^3) and pore diameter (Angstrom).

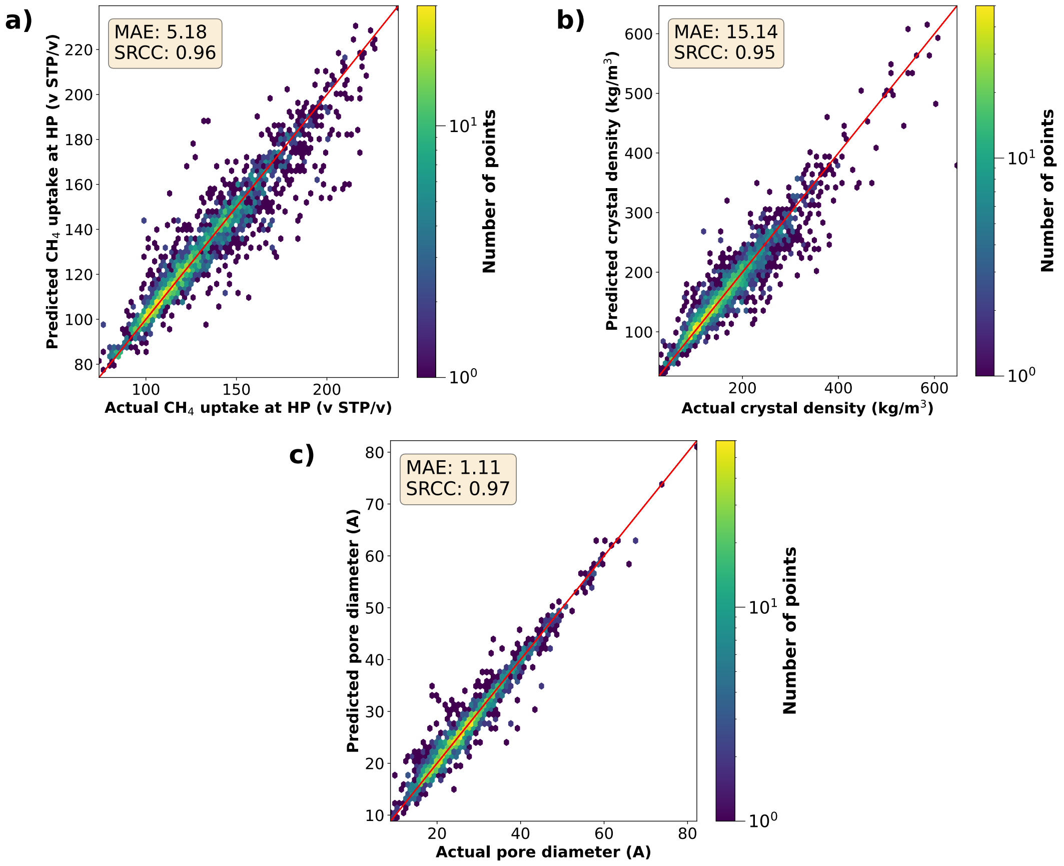

Figure S. 21| Evaluation on our model on covalent-organic frameworks (COFs) on geometric properties such as (a) methane uptake at high pressure, (b) crystal density and (c) pore diameter.[39?] It should be noted that only powder x-ray diffraction (PXRD) patterns are inputted into the model, as the precursors for COFs cannot be generated due to no metal type. Source data are provided as a Source Data file.

13 t-SNE map for MD/DFT labels

From using the actual labels of the MOF properties used for the recommendation system, it was possible to construct a t-SNE map to compare the flagging done on recommendation versus the actual recommendations through MD/DFT calculations. It can be observed that in Figure S 22, there are not many flagged MOFs; this is because there are many MOF entries in which DFT calculations do not exist; for instance, for the MOFs from QMOF, the labels for methane uptake at high pressure (for methane storage) is not available, whereas the ML map is able to make predictions on the QMOF entries for methane uptake at high pressure.

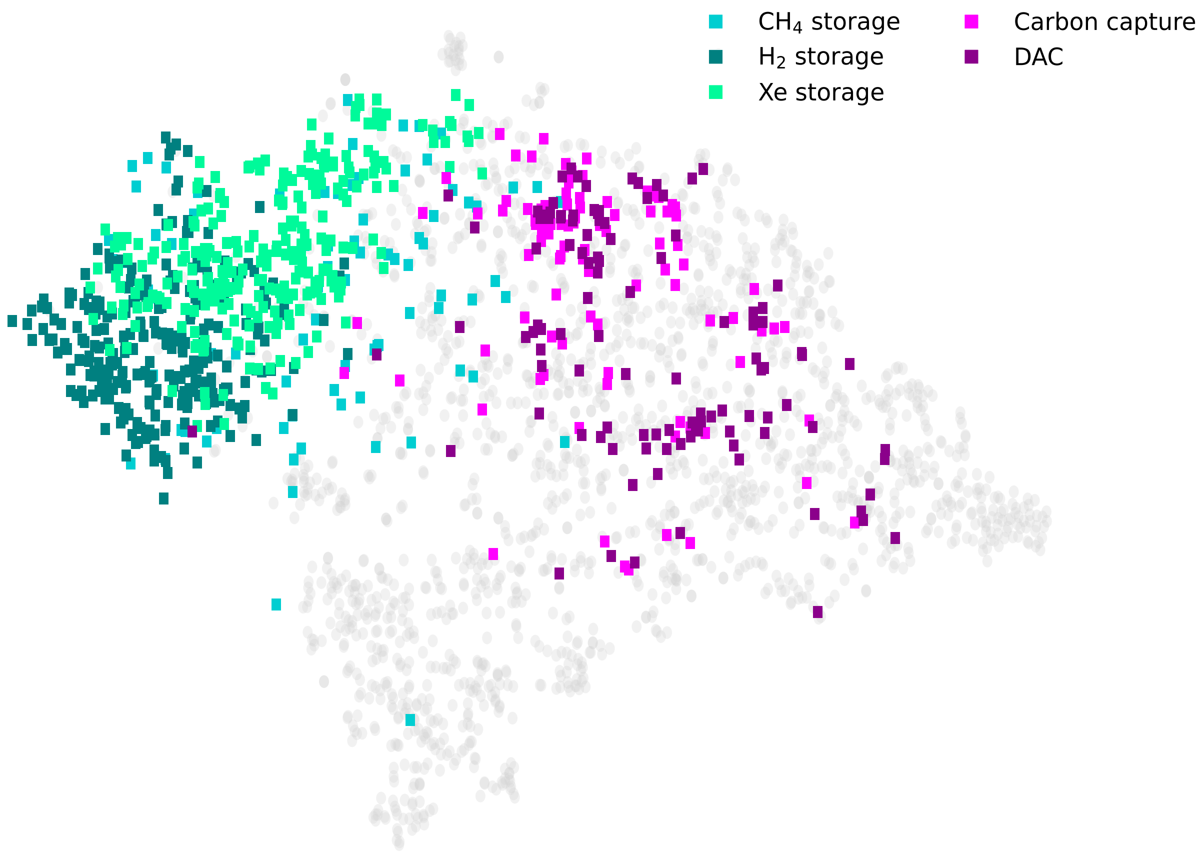

Figure S. 22| Showcasing t-distributed stochastic neighbor embedding (t-SNE) embedding space for the model space showcased in Figure 5 of the main text, but flagging is done based on available molecular simulation labels.[1,3,7] This shows that the trend of flagging metal-organic frameworks (MOFs) using our recommendation model is relatively consistent in comparison to the ground truth labels (these scatter points). Source data are provided as a Source Data file.

14 Data efficiency

Constructing labelled datasets in MOFs is often difficult due to the time-consuming and expensive process of doing experiments. Furthermore, while it is possible to retrieve labels through computational approaches such as DFT, constructing the CIF file and computationally removing the solvents from the MOF itself is time-consuming and complex. As a result, it is ideal for a model to perform well with limited data. For evaluation purposes, a small database such as ARABG (with around 400 entries) was used to demonstrate the efficiency of the model.

To showcase the impact of self-supervised learning, we wanted to look at two cases:

1. Comparison between SSL-pretrained model and scratch model;
2. Comparison between SSL-pretrained model and other benchmark models (XGB-descriptor, MOFormer, CGCNN).

Figure S 23 shows the learning curve for small data regimes. To construct the learning curve, the full dataset was split into five different folds via k-CV (k = 5), to construct the train and test sets across 5 folds to retrieve the uncertainties. It can be seen that for a chemistry-reliant property such as carbon dioxide uptake at 0.15 bar, the pretrained model performs far better than the scratch for the smallest available train set size (10 percent of train set size). While the performance of the scratch and pretrained model converges for higher data regimes, not only does it achieve the objective of performing well with limited data, but there is a general improvement in the model performance (when pretrained) until 300 data points are used as the train set. Furthermore, while there isn't a significant improvement in model performance for geometric property predictions as they are already quite satisfactory, the model performance for chemistry-reliant properties such as carbon dioxide uptake at 0.15 bar improved after pretraining against the embeddings of a crystal graph convolutional neural network (CGCNN). While the embeddings of a CGCNN provide structural information of

the MOF, it also gives information on the chemical composition, atomic information and successfully captures the local environment (bond angles, bond lengths, etc.).[11]

When comparing the efficiency of our pretrained model to a model that accepts chemistry RACs and geometric descriptors (descriptor ML), MOFormer (precursors) and CGCNN, our model exceeds these models at small data regimes. It can be seen that for small data regimes, our model exceeds the performance of all of the benchmarked models - showcasing the value of SSL-pretraining. As the training set size approaches 300 (i.e. 95 percent of the total train set size), the XGB-descriptor and CGCNN start to converge with our model performance.

Overall, the positive performance by the pretrained XRayPro showcases the importance of pretraining - especially when handling small datasets such as ARABG-DB on chemistry-reliant properties such as gas uptake at low pressure. However, it is important to note that when fine-tuning on large databases such as CoRE-MOF, BW20K, QMOF and hMOF, there is very negligible model performance improvement.

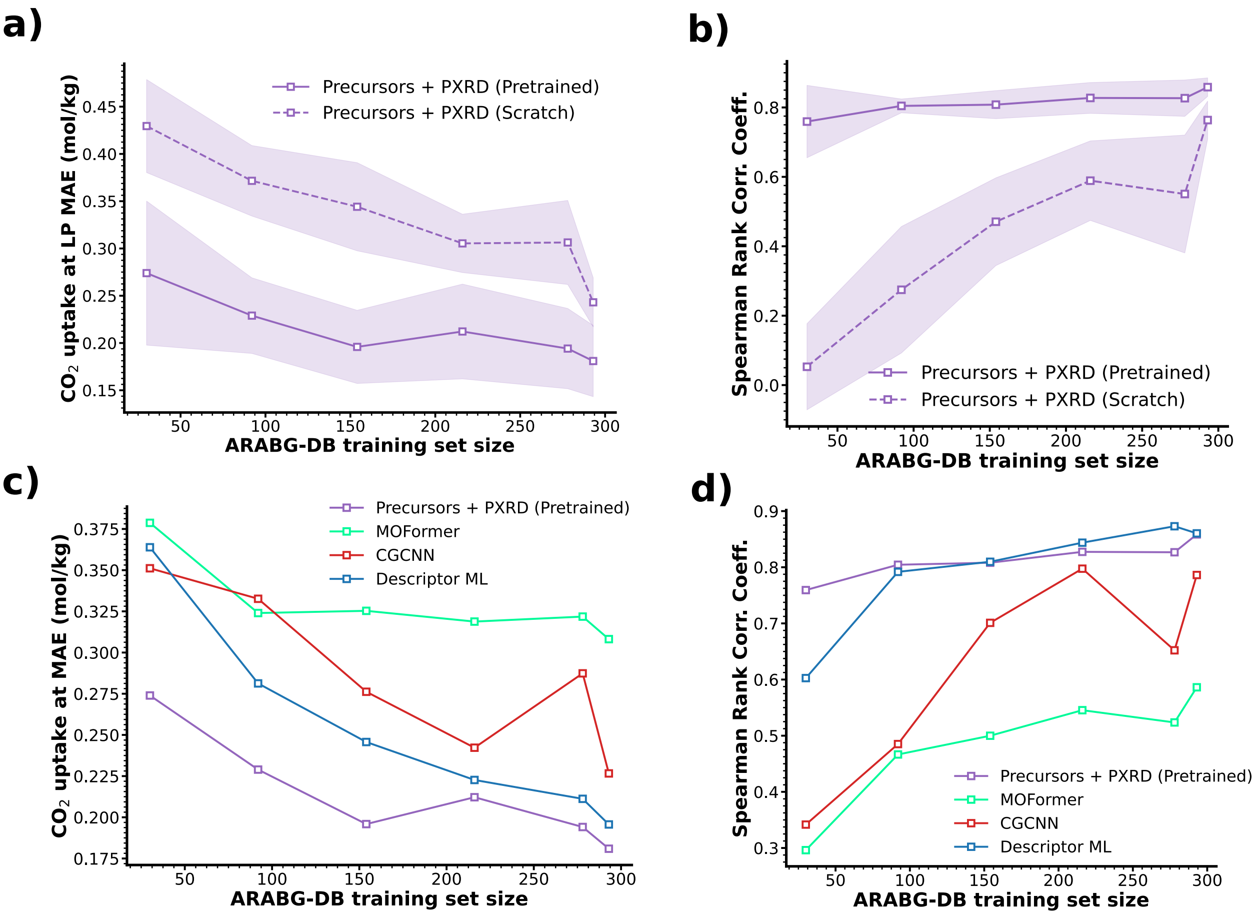

Figure S. 23| Learning curves showcasing impact of self-supervised learning (SSL) on a small dataset A comparison was made between the pretrained and scratch models of our model (panels (a) and (b) showcasing mean absolute error (MAE) and spearman rank correlation coefficient (SRCC) respectively), showcasing its influence on small datasets - particularly in very small data regimes. Furthermore, comparisons were made between benchmarked models such as a transformer-based model accepting a text-form representation of a MOF (MOFormer[9,10]), a crystal graph convolutional neural network (CGCNN[11]), descriptors such as revised autocorrelations - RACs and geometric descriptors[3] - panels (c) and (d)) and our pretrained model, showcasing its advantage at low data regimes on small datasets such as ARABG-DB.[3] Source data are provided as a Source Data file.

15 Simulating powder x-ray diffraction patterns

The CIF files from each database (CoRE-2019, BW20K, ARABG, QMOF, hMOF)[1,4,6,7] were collected and used to compute the PXRDs for each MOF through the use of Pymatgen's XRD module[40] from 0 degrees to 90 degrees. The XRD module first gets all reciprocal points in the limiting sphere, and computes Bragg's Law for each point by using:

$$\frac{\lambda}{2d_{hkl}} = \sin(\theta) \tag{6}$$

From this, the atomic scattering factors are summed over the total number of atoms in the MOF:

$$s = \frac{\sin(\theta)}{\lambda} \quad \rightarrow \quad f(s) = Z - 41.78s^2 \sum_{i=1}^{N} a_i e^{-b_i s^2} \tag{7}$$

The structure factor can then be computed:

$$F_{hkl} = \sum_{j=1}^{N} f_j \left(\frac{\sin(\theta_{hkl})}{\lambda} \right) e^{2\pi i g_{hkl} \cdot r_j} \tag{8}$$

The intensity for each plane can then be calculated by the following expression:

$$I_{hkl} = |F_{hkl}|^2 \, P(\theta) \tag{9}$$

Where $P(\theta)$ is the Lorentz polarization correction factor, given as

$$P(\theta) = \frac{1 + \cos^2(2\theta)}{\sin^2(2\theta)\cos(\theta)} \tag{10}$$

It should be noted that for Pymatgen, the Debye-Waller factor (for temperature) and the multiplicity factor are not accounted for. Furthermore, CuKa radiation was used, with no refinement on the structures done.[40,41]

16 Transformation of simulated powder x-ray diffraction patterns

When a PXRD is collected, there are usually varying number of data points. When machine learning is done, the inputs need to all be of the same length. Furthermore, if interpolation of intensity is done to fill in the missing intensities such as the 1D array of intensities can all be some fixed length, regular interpolation will result in broad peaks that do not resemble experimental PXRDs. For the collected PXRD data to resemble experimental PXRDs, transformation of the PXRD data from the initially collected sample (with some length of angles and intensities) to a 1D array of length N such that the peaks resemble the same shape as those in experimental data. The total desired size of the 1D PXRD vector is 9000, with the desired peak width being controlled by $\sigma = 0.1$. As a result, the new angle vector can be defined as:

$$2\theta_{\text{new}} = \text{linspace}(2\theta_{\text{Lower}}, 2\theta_{\text{Upper}}, 9000) \tag{11}$$

The new 1D array of intensity values for the PXRD can be initialized as $I_{\text{new}} = \text{zeros}(9000)$. While iterating through the PXRD data, for some row "i", if the simulated intensity is greater than 0:

$$I_{\text{new}} \mathrel{+}= g(2\theta_{\text{new}}, \mu = 2\theta_i, \sigma)\, I_i \tag{12}$$

Where $g(X, \mu, \sigma)$ is the Gaussian function. The 1D vector (of size 9000) I_{new} is the PXRD input into the model. Gaussian transformation is performed because peaks are realistically not perfect peaks with no peak width; Gaussian simulates this width perfectly. A summary of the Gaussian transformation of the PXRD pattern can be summarized in Algorithm 1.

Algorithm 1 Gaussian Transformation of Intensity Data

Input: Two arrays, 2θ and intensity. Combine them into a DataFrame, `data`.
Initialize: `total_points` $\leftarrow 9000$, `sigma` $\leftarrow 0.1$
`x_transformed` $\leftarrow$ `linspace`($2\theta_{\text{lower\_bound}}, 2\theta_{\text{upper\_bound}}$, `total_points`)
`y_transformed` $\leftarrow$ `zeros`(`total_points`)
for `index, row` in `data[data['intensity'] > 0].iterrows()` **do**
    `y_transformed` += `gaussian`(`x_transformed`, `row['2theta']`, `sigma`) $\times$ `row['intensity']`
end for
`y_transformed` $\leftarrow$ `y_transformed/max`(`y_transformed`) ▷ Normalization
Return: `y_transformed` ▷ This is the final transformed PXRD array

17 Generation of MOF precursors

The metal nodes and the SMILES of the organic linker were previously given for MOFs across various databases.[1,4,6,7] The chemical precursors were constructed in the format of: [Metal Type].[Organic Linker].

18 Powder x-ray diffraction pattern quality analysis

An analysis was done to assess the robustness of the model on different forms of noise and to see at what point the model robustness fails. Two scenarios were considered:

1. Low crystallinity materials – In cases where the material exhibits broad peaks due to low crystallinity, we introduced Gaussian noise to the PXRD data to simulate these distortions. The modified PXRD, denoted as C'_{PXRD}, is computed as:

$$C'_{\text{PXRD}} = C_{\text{PXRD}} + \epsilon |N(\mu = 1, \sigma^2 = 1)| \tag{13}$$

   where $N(\mu, \sigma^2)$ represents a Gaussian distribution with mean μ and variance σ^2, and ϵ is a "noise factor" ranging from 0 to 1.

2. Impure phases – To mimic situations where the synthesized material is not phase pure, we artificially introduced an additional MOF's PXRD pattern into the original

material's PXRD. The modified PXRD (C'_{PXRD}) can be expressed as:

$$C'_{\mathrm{PXRD}} = C_{\mathrm{PXRD}} + \epsilon C_{\mathrm{ref}} \quad (14)$$

where C_{ref} and ϵ are the reference PXRD and a "noise factor" ranging from 0 to 1.

Figure S 24 shows the F1-scores for the model recommendation classifications (i.e. how accurately the model can correctly predict the application for methane storage), while increasingly adding more noise. For the first case study, the model is shown to be quite robust until around 5-10 percent of Gaussian noise is added, in which the model robustness rapidly decreases and flattens out at F1-scores close to 0. For the second case study, however, we can see that the model robustness is quite acceptable even at 20 percent noise added. However, from there onwards, the performance gradually decreases - although not at a similar rate as the first case study.

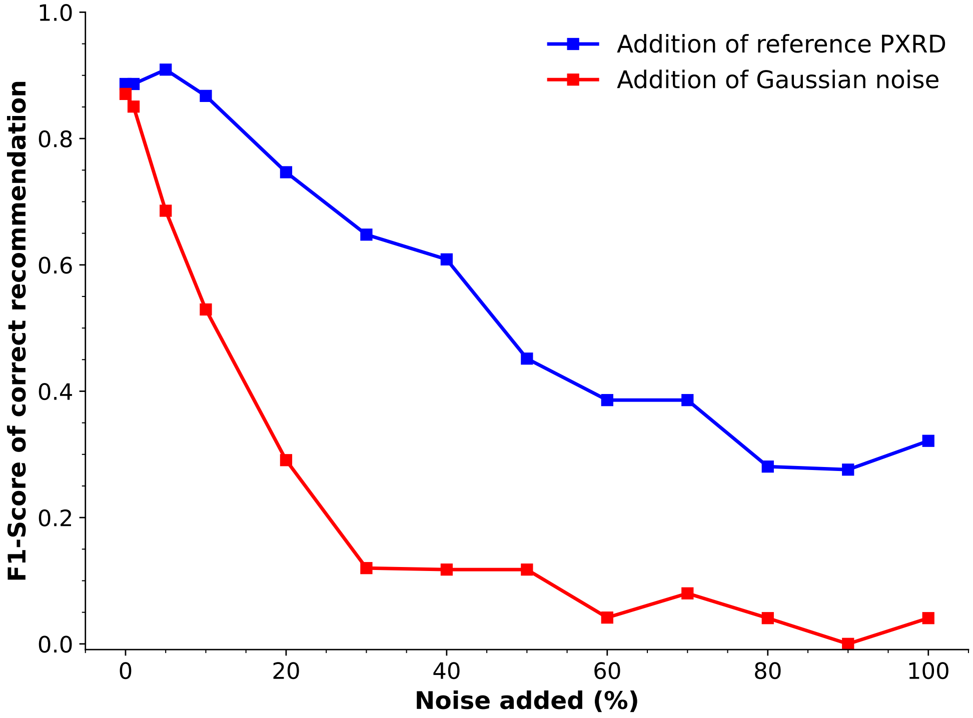

Figure S.24| F1-scores for the impact of noise in powder x-ray diffraction (PXRD) data on model robustness The two case studies looked at are when Gaussian noise is added (red) and when a reference PXRD (Cambridge Structural Database (CSD)[21] reference code: NUHQOY[42]) was added to the PXRD data (blue). Source data are provided as a Source Data file.

References

(1) Chung, Y. G.; Haldoupis, E.; Bucior, B. J.; Haranczyk, M.; Lee, S.; Zhang, H.; Vogiatzis, K. D.; Milisavljevic, M.; Ling, S.; Camp, J. S.; Slater, B.; Siepmann, J. I.; Sholl, D. S.; Snurr, R. Q. Advances, updates, and analytics for the computation-ready, experimental metal–organic framework database: CoRE MOF 2019. *Journal of Chemical & Engineering Data* **2019**, *64*, 5985–5998.

(2) Jablonka, K. M. mofchecker: A Python package for checking the validity of metal-organic framework structures. `https://github.com/kjappelbaum/mofchecker`, 2021; GitHub repository.

(3) Moosavi, S. M.; Nandy, A.; Jablonka, K. M.; Ongari, D.; Janet, J. P.; Boyd, P. G.; Lee, Y.; Smit, B.; Kulik, H. Understanding the diversity of the metal-organic framework ecosystem. *Nature Communications* **2020**, *11*, 4068.

(4) Boyd, P. G.; Woo, T. K. A generalized method for constructing hypothetical nanoporous materials of any net topology from graph theory. *CrystEngComm* **2016**, *18*, 3777–3792.

(5) Boyd, P. G. et al. Data-driven design of metal–organic frameworks for wet flue gas CO2 capture. *Nature* **2019**, *576*, 253–256.

(6) Rosen, A. S.; Iyer, S. M.; Ray, D.; Yao, Z.; Aspuru-Guzik, A.; Gagliardi, L.; Notestein, J. M.; Snurr, R. Q. Machine learning the quantum-chemical properties of metal–organic frameworks for accelerated materials discovery. *Matter* **2021**, *4*, 1578–1597.

(7) Wilmer, C. E.; Leaf, M.; Lee, C. Y.; Farha, O. K.; Hauser, B. G.; Hupp, J. T.; Snurr, R. Q. Large-scale screening of hypothetical metal–organic frameworks. *Nature chemistry* **2012**, *4*, 83–89.

(8) Chitturi, S. R.; Ratner, D.; Walroth, R. C.; Thampy, V.; Reed, E. J.; Dunne, M.; Tassone, C. J.; Stone, K. H. Automated prediction of lattice parameters from X-ray powder diffraction patterns. *Journal of Applied Crystallography* **2021**, *54*, 1799–1810.

(9) Cao, Z.; Magar, R.; Wang, Y.; Barati Farimani, A. Moformer: self-supervised transformer model for metal–organic framework property prediction. *Journal of the American Chemical Society* **2023**, *145*, 2958–2967.

(10) Bucior, B. J.; Rosen, A. S.; Haranczyk, M.; Yao, Z.; Ziebel, M. E.; Farha, O. K.; Hupp, J. T.; Siepmann, J. I.; Aspuru-Guzik, A.; Snurr, R. Q. Identification schemes for metal–organic frameworks to enable rapid search and cheminformatics analysis. *Crystal Growth & Design* **2019**, *19*, 6682–6697.

(11) Xie, T.; Grossman, J. C. Crystal graph convolutional neural networks for an accurate and interpretable prediction of material properties. *Physical review letters* **2018**, *120*, 145301.

(12) Magar, R.; Wang, Y.; Barati Farimani, A. Crystal twins: self-supervised learning for crystalline material property prediction. *npj Computational Materials* **2022**, *8*, 231.

(13) Zbontar, J.; Jing, L.; Misra, I.; LeCun, Y.; Deny, S. Barlow twins: Self-supervised learning via redundancy reduction. International conference on machine learning. 2021; pp 12310–12320.

(14) Rosen, A. S.; Fung, V.; Huck, P.; O'Donnell, C. T.; Horton, M. K.; Truhlar, D. G.; Persson, K. A.; Notestein, J. M.; Snurr, R. Q. High-throughput predictions of metal–organic framework electronic properties: theoretical challenges, graph neural networks, and data exploration. *npj Computational Materials* **2022**, *8*, 1–10.

(15) Barthel, S.; Alexandrov, E. V.; Proserpio, D. M.; Smit, B. Distinguishing metal–organic frameworks. *Crystal growth & design* **2018**, *18*, 1738–1747.

(16) Ursueguía, D.; Díaz, E.; Ordóñez, S. Metal-Organic Frameworks (MOFs) as methane adsorbents: From storage to diluted coal mining streams concentration. *Science of The Total Environment* **2021**, *790*, 148211.

(17) Zhou, W. Methane storage in porous metal- organic frameworks: current records and future perspectives. *The Chemical Record* **2010**, *10*, 200–204.

(18) Ahmed, A.; Seth, S.; Purewal, J.; Wong-Foy, A. G.; Veenstra, M.; Matzger, A. J.; Siegel, D. J. Exceptional hydrogen storage achieved by screening nearly half a million metal-organic frameworks. *Nature communications* **2019**, *10*, 1568.

(19) Mahajan, S.; Lahtinen, M. Recent progress in metal-organic frameworks (MOFs) for CO2 capture at different pressures. *Journal of Environmental Chemical Engineering* **2022**, *10*, 108930.

(20) Zhang, X.; Jablonka, K. M.; Smit, B. Deep Learning-Based Recommendation System for Metal-Organic Frameworks (MOFs). *Digital Discovery* **2024**,

(21) Moghadam, P. Z.; Li, A.; Wiggin, S. B.; Tao, A.; Maloney, A. G.; Wood, P. A.; Ward, S. C.; Fairen-Jimenez, D. Development of a Cambridge Structural Database subset: a collection of metal–organic frameworks for past, present, and future. *Chemistry of Materials* **2017**, *29*, 2618–2625.

(22) Wang, C.; Jiao, Y.; Li, G. One three-dimensional manganese (II)-organic framework bearing hydroxylphenyl imidazole dicarboxylate ligand. *Inorganic and Nano-Metal Chemistry* **2017**, *47*, 298–301.

(23) Meng, Q.-H.; Liu, J.-L.; Long, X.; Zhang, S.; Quan, Y. A porous the-type metal-organic framework based on [Mn4Cl] 7+ clusters for selective gas sorption. *Inorganic Chemistry Communications* **2017**, *79*, 46–49.

(24) Gangu, K. K.; Maddila, S.; Mukkamala, S. B.; Jonnalagadda, S. B. Synthesis, structure, and properties of new Mg (II)-metal–organic framework and its prowess as catalyst in the production of 4 H-Pyrans. *Industrial & Engineering Chemistry Research* **2017**, *56*, 2917–2924.

(25) Ramanan, A.; Balendra Structural diversity of alkaline-earth 2, 5-thiophenedicarboxylates. *Journal of Molecular structure* **2017**, *1131*, 171–180.

(26) Wang, X.; Yan, P.; Li, Y.; An, G.; Yao, X.; Li, G. Highly efficient white-light emission and UV–visible/NIR luminescence sensing of lanthanide metal–organic frameworks. *Crystal Growth & Design* **2017**, *17*, 2178–2185.

(27) Ramanan, A.; Balendra Structural diversity of alkaline-earth 2, 5-thiophenedicarboxylates. *Journal of Molecular structure* **2017**, *1131*, 171–180.

(28) Li, Y.-P.; Zhang, L.-J.; Ji, W.-J. Synthesis, characterization, crystal structure of magnesium compound based 3, 3, 5, 5-azobenzentetracarboxylic acid and application as high-performance heterogeneous catalyst for cyanosilylation. *Journal of Molecular Structure* **2017**, *1133*, 607–614.

(29) Ma, A.; Ke, F.; Jiang, J.; Yuan, Q.; Luo, Z.; Liu, J.; Kumar, A. Two lanthanide-based metal–organic frameworks for highly efficient adsorption and removal of fluoride ions from water. *CrystEngComm* **2017**, *19*, 2172–2177.

(30) Ramanan, A.; others Structural diversity of alkaline-earth 2, 5-thiophenedicarboxylates. *Journal of Molecular structure* **2017**, *1131*, 171–180.

(31) Scott, H. S.; Shivanna, M.; Bajpai, A.; Chen, K.-J.; Madden, D. G.; Perry IV, J. J.; Zaworotko, M. J. Enhanced stability toward humidity in a family of hybrid ultramicroporous materials incorporating Cr2O72–pillars. *Crystal Growth & Design* **2017**, *17*, 1933–1937.

(32) Li, H.; Li, S.; Wang, Y.; Li, Y.; Qiao, Q. Two novel MOFs constructed by tuning the metal ions—(HDMA) 2· Cd3 (FDA) 4· DMF and (HDMA)·(HTEA)· Mn3 (FDA) 4 (DMA= dimethylamine, TEA= triethylamine, FDA= furan-2, 5-dicarboxyl acid, and DMF= N, N-dimethylformamide). *Inorganic and Nano-Metal Chemistry* **2017**, *47*, 1186–1189.

(33) Hazra, A.; Jana, S.; Bonakala, S.; Balasubramanian, S.; Maji, T. K. Separation/purification of ethylene from an acetylene/ethylene mixture in a pillared-layer porous metal–organic framework. *Chemical Communications* **2017**, *53*, 4907–4910.

(34) Zhai, D.; Sun, W.; Fan, F.; Liao, X.; Chen, S.; Yang, X. Three coordination polymers constructed from 5-(4-(tetrazol-5-yl) phenyl) isophthalic acid: synthesis, crystal structure and properties. *Journal of Molecular Structure* **2017**, *1133*, 236–243.

(35) Dong, Y.; Liu, W.; Jin, J. A new rtl-type Cd (II) coordination polymer: Synthesis, structure, luminescent property, and topology analysis. *Inorganic and Nano-Metal Chemistry* **2017**, *47*, 148–152.

(36) Luo, J.; Liu, B. S.; Cao, C.; Wei, F. Neodymium (III) organic frameworks (Nd-MOF) as near infrared fluorescent probe for highly selectively sensing of Cu2+. *Inorganic Chemistry Communications* **2017**, *76*, 18–21.

(37) Suzuki, Y.; Hino, H.; Hawai, T.; Saito, K.; Kotsugi, M.; Ono, K. Symmetry prediction and knowledge discovery from X-ray diffraction patterns using an interpretable machine learning approach. *Scientific reports* **2020**, *10*, 21790.

(38) Pougin, M. J.; Domingues, N. P.; Uran, F. P.; Ortega-Guerrero, A.; Ireland, C. P.; Espín, J.; Lee Queen, W.; Smit, B. Adsorption in Pyrene-Based Metal–Organic Frameworks: The Role of Pore Structure and Topology. *ACS Applied Materials & Interfaces* **2024**, *16*, 36586–36598.

(39) Jablonka, K. M.; Ongari, D.; Moosavi, S. M.; Smit, B. Big-data science in porous materials: materials genomics and machine learning. *Chemical reviews* **2020**, *120*, 8066–8129.

(40) Ong, S. P.; Richards, W. D.; Jain, A.; Hautier, G.; Kocher, M.; Cholia, S.; Gunter, D.; Chevrier, V.; Persson, K. A.; Ceder, G. Python Materials Genomics (pymatgen): A Robust, Open-Source Python Library for Materials Analysis. *Computational Materials Science* **2013**, *68*, 314–319.

(41) Graef, M. D.; McHenry, M. E. *Structure of Materials: An Introduction to Crystallography, Diffraction, and Symmetry*; Cambridge University Press: Cambridge, UK, 2007.

(42) Xing, S.; Zeng, G.; Liu, X.; Yang, F.; Hao, Z.; Gao, W.; Yang, Y.; Wang, X.; Li, G.; Shi, Z.; Feng, S. Multifunctional luminescence properties of co-doped lanthanide metal organic frameworks. *Dalton Transactions* **2015**, *44*, 9588–9595.
